# FAS-associated factor-1 positively regulates type I interferon response to RNA virus infection by targeting NLRX1

**DOI:** 10.1371/journal.ppat.1006398

**Published:** 2017-05-22

**Authors:** Jae-Hoon Kim, Min-Eun Park, Chamilani Nikapitiya, Tae-Hwan Kim, Md Bashir Uddin, Hyun-Cheol Lee, Eunhee Kim, Jin Yeul Ma, Jae U. Jung, Chul-Joong Kim, Jong-Soo Lee

**Affiliations:** 1 College of Veterinary Medicine, Chungnam National University, Daejeon, Republic of Korea; 2 Faculty of Veterinary & Animal Science, Sylhet Agricultural University, Sylhet, Bangladesh; 3 College of Biological Sciences and Biotechnology, Chungnam National University, Daejeon, Korea; 4 Korean Medicine (KM)-Application Center, Korea Institute of Oriental Medicine (KIOM), Daegu, Republic of Korea; 5 Department of Molecular Microbiology and Immunology, Keck School of Medicine, University of Southern California, California, United States of America; University of North Carolina at Chapel Hill, UNITED STATES

## Abstract

FAS-associated factor-1 (FAF1) is a component of the death-inducing signaling complex involved in Fas-mediated apoptosis. It regulates NF-κB activity, ubiquitination, and proteasomal degradation. Here, we found that FAF1 positively regulates the type I interferon pathway. FAF1^gt/gt^ mice, which deficient in FAF1, and FAF1 knockdown immune cells were highly susceptible to RNA virus infection and showed low levels of inflammatory cytokines and type I interferon (IFN) production. FAF1 was bound competitively to NLRX1 and positively regulated type I IFN signaling by interfering with the interaction between NLRX1 and MAVS, thereby freeing MAVS to bind RIG-I, which switched on the MAVS-RIG-I-mediated antiviral signaling cascade. These results highlight a critical role of FAF1 in antiviral responses against RNA virus infection.

## Author summary

Type I interferon-mediated antiviral response is critical for controlling virus infections. However, interferon-mediated immune responses need to be tightly regulated to maintain host immune homeostasis. Recently, molecules involved in regulating interferon-mediated innate immune response are the subject of much research. Among these, the first protein to be identified as a negative regulator of MAVS was the nucleotide-binding domain and leucine-rich repeat containing family member, NLRX1. NLRX1 associates with MAVS to inhibit antiviral signaling by interrupting virus-induced RLR-MAVS interactions. Interestingly, we found that FAF1 interacts with NLRX1 in response to RNA virus infection and this interaction inhibits binding of MAVS to NLRX1, which in turn switches on RIG-I mediated antiviral immune responses. As results, we showed that FAF1^gt/gt^ mice, which deficient in FAF1, and FAF1 knockdown immune cells were highly susceptible to RNA virus infection and showed low levels of inflammatory cytokines and type I interferon (IFN) production. Our findings suggest that FAF1 is a crucial regulator that induces the antiviral innate immune responses against RNA virus infection.

## Introduction

FAS-associated factor 1 (FAF1) was originally identified as a member of the FAS death-inducing signaling complex [1]. FAF1 harbors several protein interaction domains, including FAS-interacting domains (FID), a death effector domain-interacting domain (DEDID), and multi-ubiquitin-related domains, which interact with ubiquitinated target proteins and regulate their proteolysis [2]. Although FAF1 initially demonstrated to have Fas induced apoptotic potential [3], it also has diverse biological functions such as regulation of NF-κB signaling, chaperone activity and proteosomal degradation by ubiquitination. [2,4–7].

Early recognition of invading viruses by host cells is critical to antiviral innate immunity. Invading viruses trigger type I interferon-mediated antiviral responses and induce production of effector proteins that inhibit completion of the virus cycle and virus dissemination in vivo [8–12]. Germline-encoded pattern recognition receptors (PRRs) within the innate immune system sense signature molecules expressed by pathogens, known as pathogen-associated molecular patterns (PAMPs). To date, PRRs are classified into three families: retinoic acid inducible gene (RIG)-I-like receptors (RLRs), Toll-like receptors (TLR), and the nucleotide oligomerization domain (NOD) and leucine-rich repeat and pyrin domain-containing (NLRP) proteins [8,13]. RLRs such as RIG-I and melanoma differentiation-associated gene-5 (MDA-5) are important molecules that detect viral RNA in the cytosol. In uninfected cells, RIG-I exists in an auto-repressed conformation in which the caspase activation and recruitment domains (CARDs) are not available for binding to induce downstream signal transduction [14]. Upon recognition of viruses, particularly RNA viruses, RIG-I is activated and undergoes self-dimerization and structural modifications that permit CARD-CARD interactions with the downstream adapter molecule, mitochondrial antiviral signaling protein (MAVS; also known as IPS-1, VISA, and Cardif) [15–20]. Then it activates type I interferon responses via downstream signaling molecules TBK1/IKKi and IRF3, and NF-κB activation via IKK, to elicit inflammatory responses [21–26].

However, interferon- or NF-κB-mediated immune responses need to be tightly regulated to maintain host immune homeostasis, otherwise the uncontrolled immune response can be deleterious, or even fatal, to the host [27–32]. Hence, molecules involved in regulating interferon-mediated innate immune response are the subject of much research. Indeed, mechanisms that regulate RIG-I-mediated antiviral signaling, which is tightly controlled by a series of positive and negative regulators, have been reported [13,33,34]. Among these, NLRX1, a member of the nucleotide-binding domain and leucine-rich-repeat-containing (NLR) protein family, resides on the outer mitochondrial membrane and interfere CARD-CARD interactions between MAVS and RIG-I to negatively regulate antiviral interferon signaling [35–38]. However, during virus infection, the mechanism which controls type I interferon (IFN) signaling via modulating the MAVS and NLRX1 interaction, needs to be investigated more in detail. Here, we show that FAF1 is a positive regulator of the NF-κB and type I interferon signaling pathways during RNA virus infection. FAF1 competitively binds to NLRX1, thereby disrupting its interaction with MAVS and ultimately amplifying the downstream antiviral immune response.

## Results

### FAF1^gt/gt^ mice show increased susceptibility to virus infection

**Fig 1:**
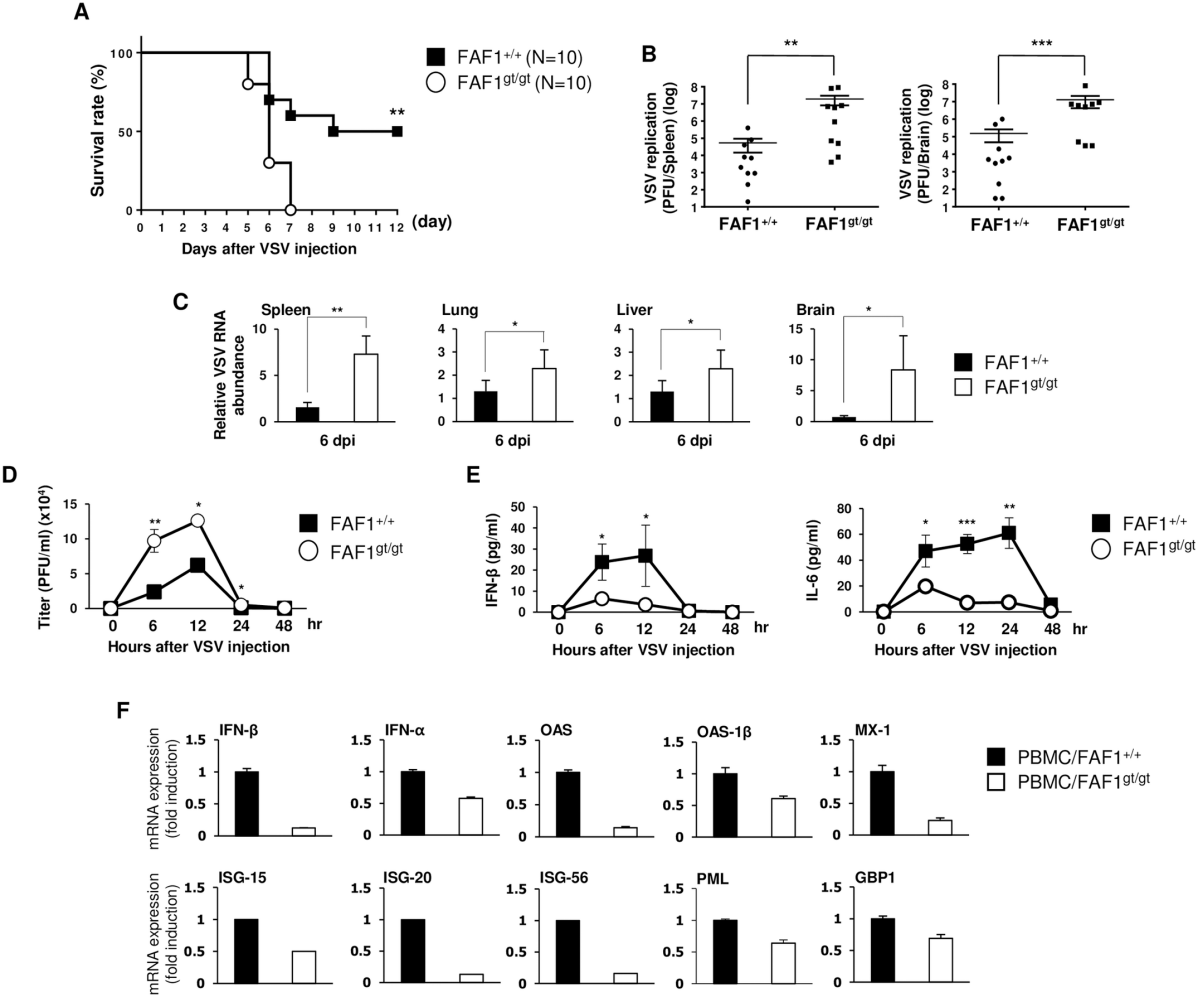
FAF1^gt/gt^ mice are susceptible to virus infection and show suppressed immune responses. (A) Wild-type mice (FAF1^+/+^) (n = 10) and FAF1 knockdown mice (FAF1^gt/gt^) (n = 10) were infected with VSV-Indiana (2 × 10^8^ pfu/mouse) via tail-vein injection and survival was monitored for 12 days. (B) Organs (spleen and brain) from FAF1^+/+^ (n = 10) and FAF1^gt/gt^ (n = 10) mice were collected at 6 dpi with VSV-Indiana (2 × 10^8^ pfu/mouse) via tail-vein injection. Virus titers in supernatants of homogenized tissues were measured by plaque assay. (C) The viral load in supernatants of homogenized spleen, lung, liver, and brain tissues from FAF1^+/+^ (n = 4) and FAF1^gt/gt^ (n = 4) mice infected with VSV-Indiana (2 × 10^8^ pfu/mouse) via tail-vein injection was measured by qRT-PCR at 6 dpi. (D and E) FAF1^+/+^ (n = 10) and FAF1^gt/gt^ (n = 10) mice were infected with VSV-GFP (4 × 10^8^ pfu/mouse) via tail-vein injection. Sera were collected from the mice at indicated time points and the virus titer was determined by plaque assay. IFN- β and IL-6 were measured by ELISA. (F) PBMCs were isolated from whole peripheral blood of FAF1^+/+^ (PBMC/FAF1^+/+^; n = 5) and FAF1^gt/gt^ (PBMC/FAF1^gt/gt^; n = 5) mice infected with VSV-GFP (4 × 10^8^ pfu/mouse) via tail-vein injection. Total RNA was extracted from PBMCs at 24 hpi and used for qRT-PCR analysis to determine levels of IFN-β, IFN-α, OAS, OAS-1β, MX-1, ISG-15, ISG-20, ISG-56, PML and GBP1 mRNA. All the mRNA expressions were normalized to GAPDH. Data are presented as the mean ± SEM. *p < 0.05, **p < 0.01, ***p < 0.001 (Student’s t test or log-rank test). Data are representative of at least two independent experiments.

To examine the biological function of FAF1, we performed experiments using FAF1^+/+^ and FAF1^gt/gt^ mice after confirmed by genotyping (S1 Fig, panels A-B-C). First, mice were infected with the of vesicular stomatitis virus (VSV) Indiana strain (VSV-Indiana) via tail-vein injection and their survival was monitored to determine susceptibility to viral infection (Fig 1, panel A). Knockdown of FAF1 rendered mice significantly more susceptible to lethal VSV infection. A plaque assay and quantitative real-time polymerase chain reaction (qRT-PCR) was conducted to measure the amount of VSV in spleen, lung, liver, and brain tissues at 24 hr and 6 days post-infection (hpi and dpi) (Fig 1, panels B-C and S1 Fig, panel D). Organs from FAF1^gt/gt^ mice contained higher amount of virus than those from FAF1^+/+^ mice. This suggests that the virus replicates more actively in FAF1^gt/gt^ mice than in FAF1^+/+^ mice, resulting increased mortality. Additionally, serum samples were collected at different time points after mice were infected with green fluorescent protein (GFP)-tagged VSV (VSV-GFP) (Fig 1, panels D-E) or treated with Poly (I:C) (S1 Fig, panel E). The serum of FAF1^gt/gt^ mice contained more replicating virus and lower levels of IFN-β and IL-6 than that of FAF1^+/+^ mice, indicating that knockdown of FAF1 suppresses cytokine secretion upon virus infection. Moreover, peripheral blood mononuclear cells (PBMCs) from both groups of mice injected with VSV-GFP via tail-vein were collected and measured to check mRNA encoding IFN-related genes expression at 24 hpi (Fig 1, panel F). PBMCs from FAF1^gt/gt^ mice expressed lower levels of mRNA encoding IFN-related genes than those from FAF1^+/+^ mice. These results provide in vivo evidence that FAF1 knockdown affects type I IFN mediated signaling and antiviral immunity.

### Bone Marrow-Derived Macrophages (BMDMs), Bone Marrow-Derived Dendritic Cells (BMDCs) and PBMCs isolated from FAF1^gt/gt^ mice show reduced type I IFN signaling and are more permissive to viral replication

**Fig 2:**
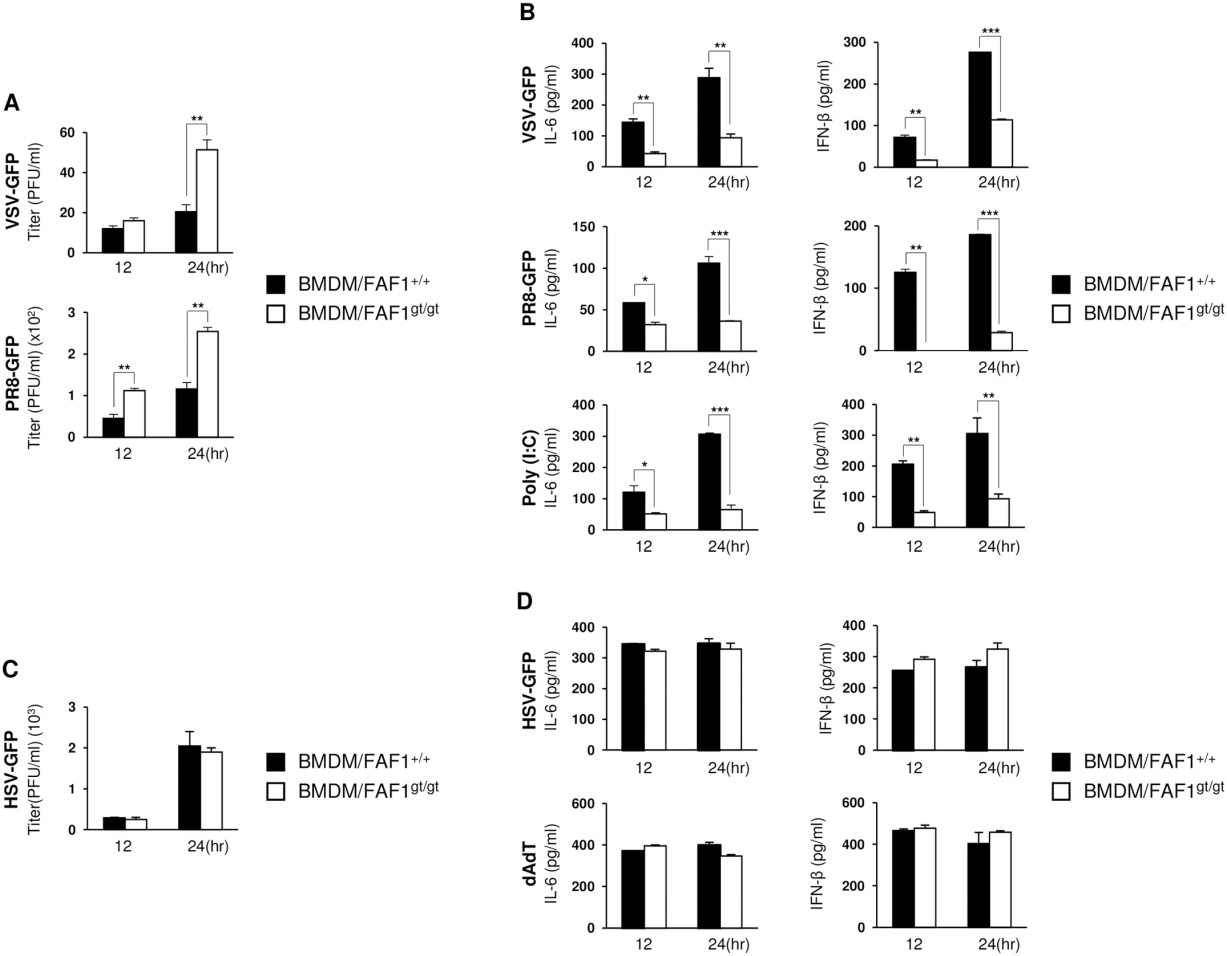
FAF1 plays a role in antiviral activity in BMDMs upon RNA virus infection. (A and B) Wild-type BMDMs (BMDM/FAF1^+/+^) or FAF1 knockdown BMDMs (BMDM/FAF1^gt/gt^) were stimulated with RNA virus (VSV-GFP (MOI = 2), PR8-GFP (MOI = 3)) or RNA stimulant (Poly (I:C) (20 μg/ml)). (C and D) BMDM/FAF1^+/+^ or BMDM/FAF1^gt/gt^ were stimulated with DNA virus (HSV-GFP (MOI = 2)) or DNA stimulant (dAdT (1 μg/ml)). Virus titers (A and C) and IL-6 or IFN-β levels (B and D) were measured by plaque assay and ELISA, respectively. Data are presented as the mean ± SEM. *p < 0.05, **p < 0.01, ***p < 0.001 (Student’s t test). Data are representative of at least two independent experiments.

BMDMs were isolated from the bone marrow of FAF1^+/+^ and FAF1^gt/gt^ mice and infected with VSV-GFP or GFP tagged H1N1 influenza virus (A/PR8/8/34; PR8-GFP). Virus replication was higher in BMDMs of FAF1^gt/gt^ than in those of FAF1^+/+^ mice at 12 and 24 hpi (Fig 2, panel A). To determine the reason for the increased viral replication in BMDMs of FAF1^gt/gt^ mice, IL-6 and IFN-β levels were analyzed after 12 and 24 hr of VSV-GFP and PR8-GFP infection or Poly (I:C) treatment (Fig 2, panel B). BMDMs of FAF1^gt/gt^ mice produced less IL-6 and IFN-β than BMDMs of FAF1^+/+^ mice. Next, BMDCs and PBMCs were isolated from FAF1^+/+^ and FAF1^gt/gt^ mice, and stimulated with VSV-GFP, PR8-GFP or Poly (I:C). Virus titers and cytokine secretion were then compared (S2 Fig). BMDCs and PBMCs isolated from FAF1^gt/gt^ mice harbored greater amounts of virus and secreted lower levels of cytokines than BMDCs and PBMCs of FAF1^+/+^ mice. These data suggest that immune cells within the BMDMs, BMDCs, and PBMCs populations from FAF1^gt/gt^ mice show inhibited type I IFN signaling, which facilitates viral replication.

To find out whether FAF1 has a similar effect after infection with a DNA virus, BMDMs were isolated from FAF1+/+ and FAF1gt/gt mice and infected with GFP tagged Herpes Simplex virus 1 (HSV-GFP) (S2 Fig, panels C-D). There was no difference in the observed levels of cytokine secretion or virus replication between BMDMs from the two groups of mice. This confirms that FAF1 has no role in DNA virus-stimulated type I IFN signaling. Taken together, these data suggest that FAF1 positively regulates type I IFN signaling in response to infection by RNA viruses.

### FAF1 knockdown suppresses type I IFN secretion and augments viral replication in murine embryonic fibroblasts (MEFs)

**Fig 3:**
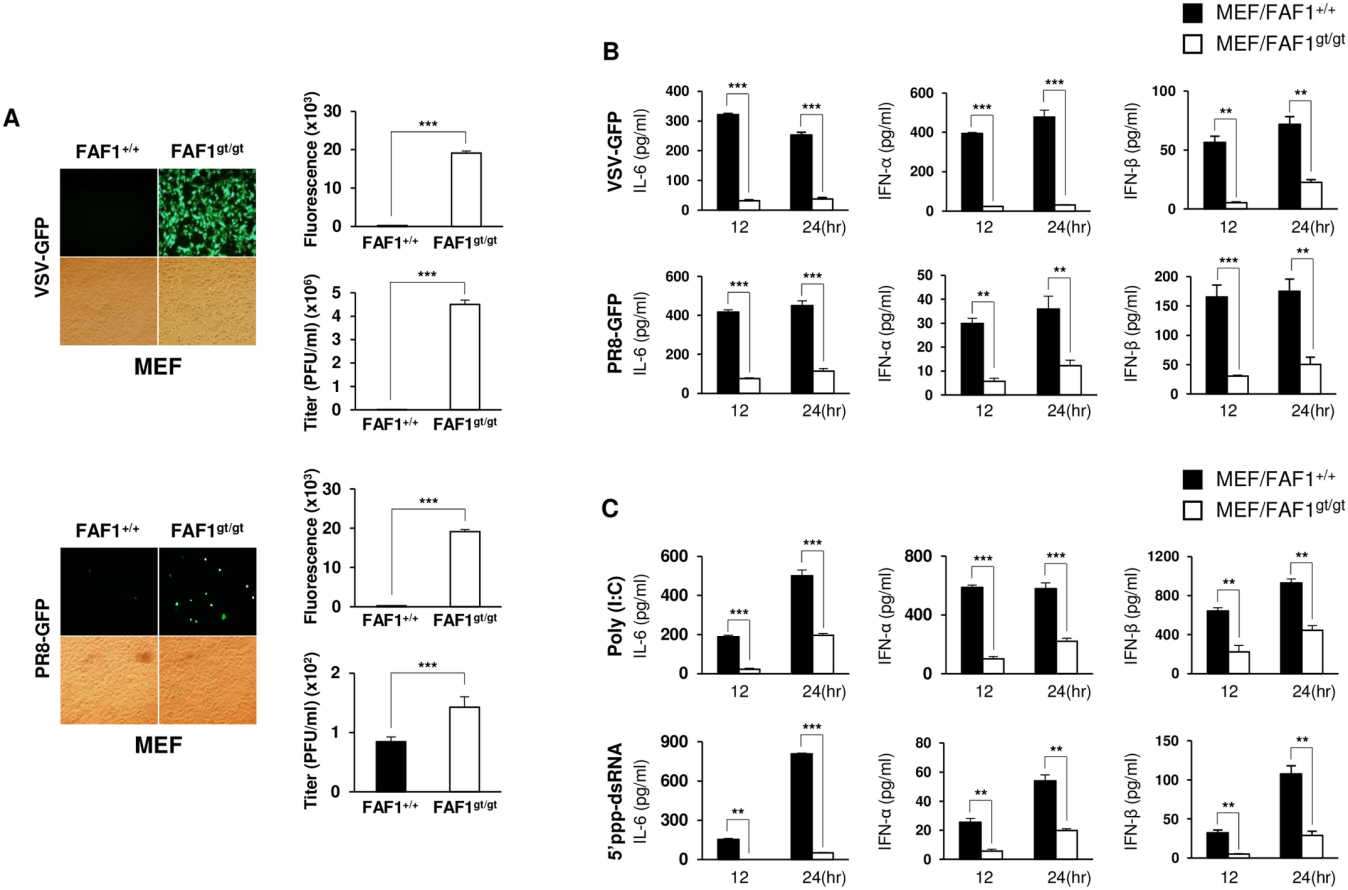
Knockdown of FAF1 augments viral replication and reduces Type I IFN secretion in MEFs. (A) Wild-type MEFs (MEF/FAF1^+/+^) and FAF1 knockdown MEFs (MEF/FAF1^gt/gt^) were infected with VSV-GFP (MOI = 0.5) or PR8-GFP (MOI = 1). GFP expression of infected cells was visualized at 24 hpi, under a fluorescence microscopy (200 × magnification) and quantified using a fluorescence modulator. Virus titers were measured by plaque assay. (B and C) MEF/FAF1^+/+^ and MEF/FAF1^gt/gt^ were infected with VSV-GFP (MOI = 0.5) or PR8-GFP (MOI = 1) (B) and treated with Poly (I:C) (20 μg/ml) or 5’ppp-dsRNA (1 μg/ml) (C). Levels of IL-6, IFN-α, and IFN-β in supernatants were measured by ELISA after 12 or 24 of infection or treatment. Data are presented as the mean ± SEM. *p < 0.05, **p < 0.01, ***p < 0.001 (Student’s t test). Data are representative of at least two independent experiments.

To examine the effect of FAF1 on virus replication *in vitro*, we prepared FAF1 knockdown MEFs from FAF1^gt/gt^ mice. FAF1 knockdown was confirmed by immunoblot analysis (S3 Fig, panel A). Virus titers and cytokine levels were measured at 12 and 24 hpi with VSV-GFP, PR8-GFP (Fig 3, panels A-B) or GFP tagged New castle disease virus (NDV-GFP) (S3 Fig, panels B-C). The amount of GFP expressed by cells following viral infection was examined by fluorescence microscopy and quantitated using a fluorescence modulator. FAF1 knockdown MEFs showed increased GFP expression. The virus titer was also higher in FAF1 knockdown MEFs than in wild-type (WT) MEFs (Fig 3, panel A and S3 Fig, panel B). Supernatants from FAF1 knockdown MEFs contained less IL-6, IFN-α, and IFN-β than those from WT MEFs (Fig 3, panel B and S5 Fig, panel C). Moreover, supernatants from FAF1 knockdown cells contained lower levels of cytokines than those from WT cells after stimulation with Poly (I:C) or 5’ppp-dsRNA (Fig 3, panel C). Taken together, these data suggest that knockdown of FAF1 inhibited the immune responses by reducing IFN secretion in response to viral infection, thereby facilitating virus replication. Additionally, FAF1-reconstituted MEFs were prepared and expression of FAF1 was confirmed by immunoblotting (S3 Fig, panel D). Virus titers and cytokine levels in FAF1 knockdown MEFs and FAF1-reconstituted MEFs were compared after virus infection (S3 Fig, panels E-F-G). FAF1-reconstituted cells showed reduced viral replication and higher cytokine secretion than FAF1 knockdown MEFs, demonstrating that reconstitution of FAF1 restores induction of type I IFN signaling.

### Knockdown and overexpression of FAF1 in RAW264.7 cells affects type I IFN secretion and viral replication

**Fig 4:**
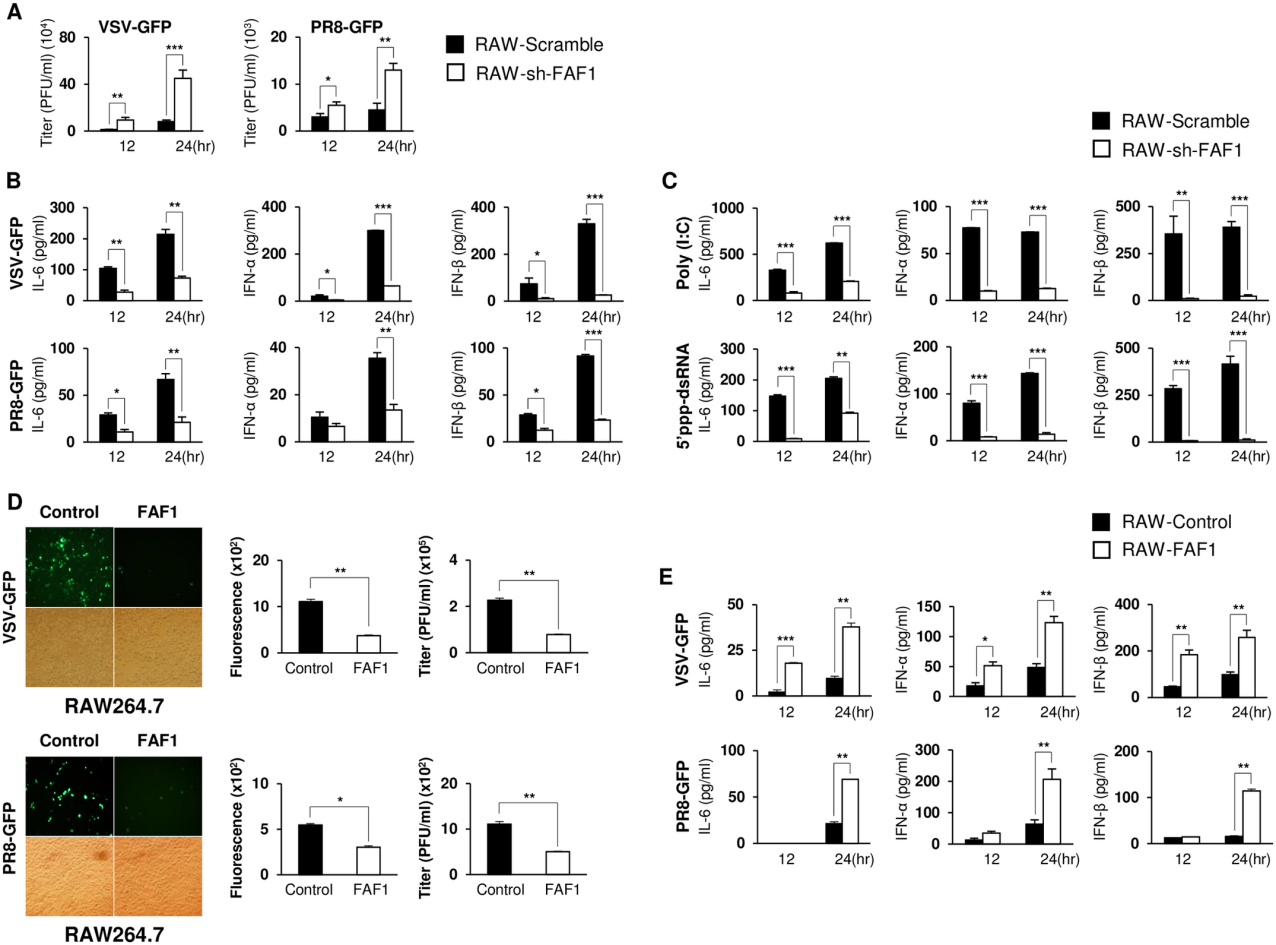
FAF1 plays a role in antiviral activity in RAW264.7 cells. (A and B) RAW264.7 cells were infected with lentivirus harboring scramble and FAF1 shRNA to prepare control RAW264.7 (RAW-Scramble) and FAF1 knockdown RAW264.7 (RAW-sh-FAF1), respectively. Cells were infected with VSV-GFP (MOI = 2) or PR8-GFP (MOI = 3). After 12 and 24 hr, the virus titer was measured by plaque assay (A) and IL-6, IFN-α, and IFN-β levels in the supernatant were measured by ELISA (B). (C) Cells were treated with Poly (I:C) (20 μg/ml) or 5’ppp-dsRNA (1 μg/ml), and levels of IL-6, IFN-α, and IFN-β in the supernatant were measured by ELISA. (D and E) RAW264.7 cells were transfected with an empty IRES vector (control) and a FAF1-containing IRES plasmid. Stably expressing control (RAW-Control) and FAF1-overexpressing (RAW-FAF1) cells were infected with VSV-GFP (MOI = 1) or PR8-GFP (MOI = 2). At 24 hpi, GFP expression was visualized under a fluorescence microscopy (200 × magnification) and quantified using a fluorescence modulator. Virus titers were measured by plaque assay (D). Culture supernatants were collected at 12 h and 24 hpi, and IL-6, IFN-α, and IFN-β levels were measured by ELISA (E). Data are presented as the mean ± SEM. *p < 0.05, **p < 0.01, ***p < 0.001 (Student’s t test). Data are representative of at least two independent experiments.

To exclude the possibility that positive regulation of type I IFN signaling by FAF1 is a cell type-specific phenomenon, knockdown FAF1 murine macrophage cell line was prepared by infecting a lentivirus harboring FAF1 shRNA (small hairpin RNAs) or transfecting FAF1 siRNA (small interfering RNA) to RAW264.7. First, reduced FAF1 expression was confirmed by immunoblot analysis (S4 Fig, panel A). Viral titers and cytokine levels were evaluated after VSV-GFP or PR8-GFP infection (Fig 4, panels A-B and S4 Fig, panels B-C) and treatment with Poly (I:C) or 5’ppp-dsRNA (Fig 4, panel C). Consistent with our previous results, viral titers were higher and cytokine levels were lower in both shRNA and siRNA FAF1 knockdown RAW264.7 cells than in control (scramble) cells. Additionally, THP-1 cells (a human immune cell line) were transfected with siRNA targeting FAF1, and virus replication and cytokine levels were measured after virus infection (S4 Fig, panels D-E-F-G). The results were similar to those for FAF1 knockdown RAW264.7 cells. To confirm these results, we generated stable FAF1 overexpressing RAW264.7 cells and overexpression was confirmed by immunoblot analysis (S5 Fig, panel A). FAF1-overexpressing RAW264.7 cells infected with PR8-GFP, VSV-GFP (Fig 4, panels D-E) or NDV-GFP (S5 Fig, panels B-C) showed lower levels of viral replication and higher levels of IL-6, IFN-β, and IFN-α production than control RAW264.7 cells. Treatment with Poly (I:C) or 5’ppp-dsRNA yielded consistent results with the virus infection experiments (S5 Fig, panel D). Additionally, to find out whether FAF1 has no effect to DNA virus infection in RAW264.7 cells, similar to HSV infection in BMDMs, GFP tagged adenovirus (Adeno-GFP) were infected to control and FAF1 knockdown (S6 Fig, panel A) or overexpressing (S6 Fig, panel B) RAW264.7 cells. Accordance with the results of HSV-GFP in BMDMs, Adeno-GFP experiment also showed no difference in virus replication and cytokine secretion levels between control and FAF1 knockdown or overexpressing cells. Taken together, these results suggest that, irrespective of the cell type, FAF1 positively regulates type I IFN secretion upon RNA virus infection, and not upon DNA virus infection.

Moreover, to confirm whether enhanced VSV-GFP replication in FAF1 knockdown RAW264.7 cells and MEFs was due to repressed IFN secretion by knockdown of FAF1 and not due to intrinsic block to replication of RNA viruses, we infected VSV-GFP to FAF1 knockdown RAW264.7 cells and MEFs in the presence of an anti-IFNAR blocking antibody (IFNAR Ab) and determined VSV-GFP replication level (S7 Fig, panels A-B). As shown in the results, IFNAR Ab treated control cells showed almost two to three times higher virus replication level compared with non-treated control cells due to the IFNAR blocking effect of IFNAR Ab. On the contrary, in FAF1 knockdown cells, virus replication levels slightly enhanced after treatment of IFNAR Ab, which indicated that IFNAR Ab could not exhibit IFNAR blocking effect prominently in FAF1 knockdown RAW264.7 cells, since type I IFN secretion was already suppressed by knockdown of FAF1. These results suggests that enhanced or reduced virus replication depends on knockdown or overexpression of FAF1 due to the regulation of type I IFN secretion by FAF1. Furthermore, from our results of Poly (I:C) and 5’ppp-dsRNA stimulation studies, we could anticipate that FAF1 regulates type I IFN signaling through RIG-I-MAVS signaling pathway, as those stimulants induce type I IFN signaling pathway by activating RIG-I. To investigate whether FAF1 regulates type I IFN secretion not through TLR7 and TLR9, we also stimulated the TLR7 and TLR9 by their agonists, imiquimod and ODN2395, respectively to FAF1 knockdown RAW264.7 cells (S7 Fig, panel C). According to data, the IL-6 and IFN-β secretion levels of control and FAF1 knockdown RAW264.7 cells were similar, which indicate that FAF1 regulates type I IFN signaling through RIG-I mediated pathway, and not via the TLR7 and TLR9 mediated pathway.

### FAF1 enhances type I IFN signaling and induces transcription of IFN-related genes

**Fig 5:**
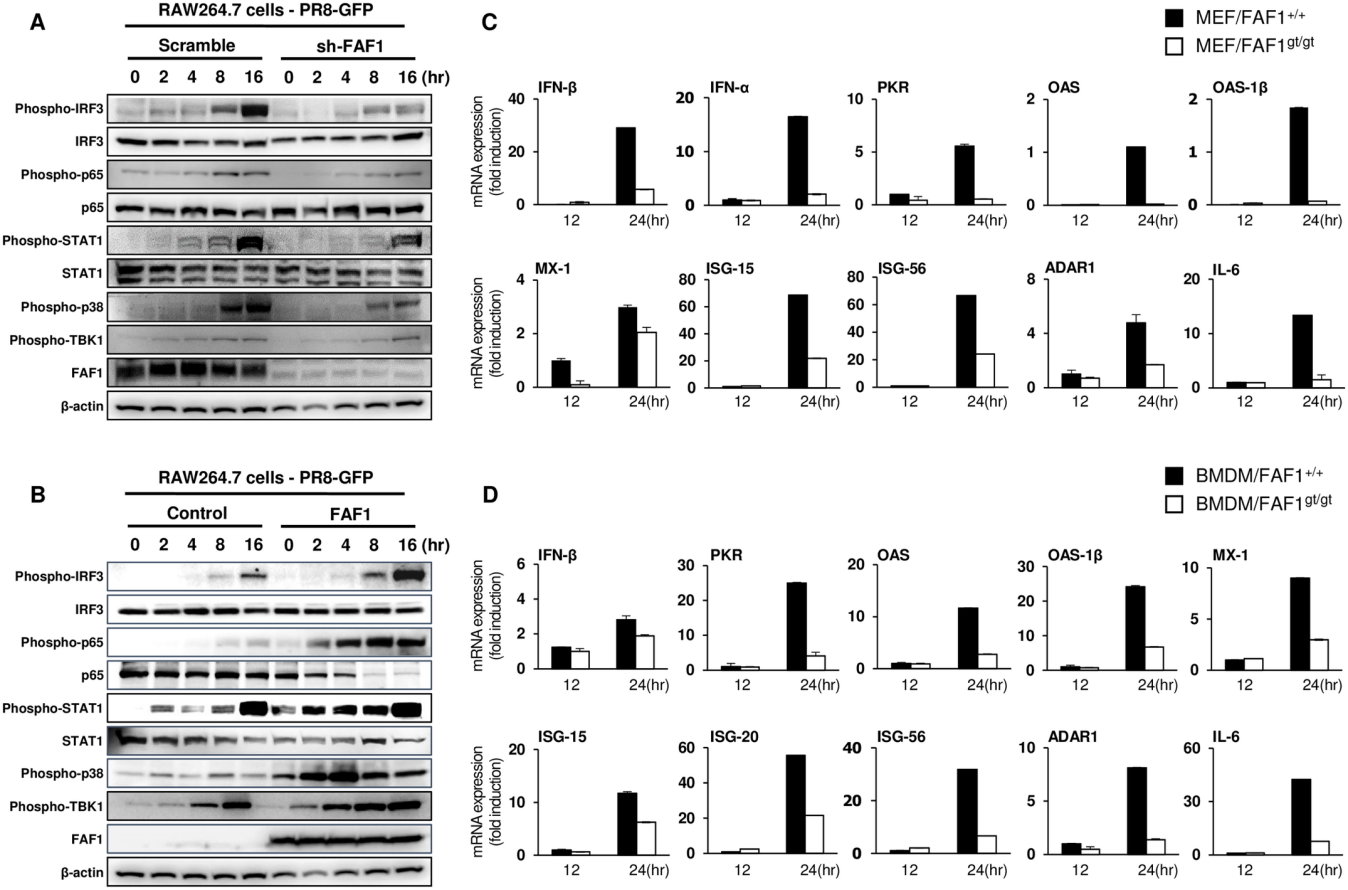
FAF1 activates the Type I IFN signaling pathway and induces IFN-related gene expression. (A and B) Control RAW264.7 (RAW-Scramble) and FAF1 knockdown RAW264.7 (RAW-sh-FAF1) cells (A) or control RAW264.7 (RAW-Control) and FAF1-overexpressing RAW264.7 (RAW-FAF1) cells (B) were infected with PR8-GFP (MOI = 2). At the indicated time points after infection, phosphorylated IRF3, p65, STAT1, p38 and TBK1, and total IRF3, p65 and STAT1 were measured in cell extracts by immunoblotting. β-actin was used to confirm equal loading of proteins. (C and D) Wild-type MEFs (MEF/FAF1^+/+^) and FAF1 knockdown MEFs (MEF/FAF1^gt/gt^) (C) and BMDMs isolated from FAF1^+/+^ (BMDM/FAF1^+/+^) and FAF1^gt/gt^ (BMDM/FAF1gt/gt) mice (D) were infected with PR8-GFP (MOI = 1 and 3, respectively) for 12 hr, followed by total RNA extraction. Expression of mRNA encoding IFN-β, IFN-α, PKR, OAS, OAS-1β, MX-1, ISG-15, ISG-56, ADAR1 and IL-6 for MEFs and IFN-β, PKR, OAS, OAS-1β, MX-1, ISG-15, ISG-20, ISG-56, ADAR1 and IL-6 for BMDMs was analyzed by qRT-PCR. Data are presented as the mean ± SEM. Data are representative of at least two independent experiments.

To further examine the effects of FAF1 on the antiviral signaling cascade, we next examined virus-induced phosphorylation of IRF3, p65, STAT1, p38, and TBK1. Cells were stimulated with PR8-GFP and samples were collected at indicated time points. Whole cell lysates (WCL) were prepared and analyzed by immunoblotting (Fig 5, panels A-B and S8 Fig, panel A). First, scramble and FAF1 knockdown RAW264.7 cells were infected with PR8-GFP, and phosphorylation levels of the indicated proteins were examined (Fig 5, panel A). Protein phosphorylation in both scramble and FAF1 knockdown RAW264.7 cells was initiated at 8 hpi, and increased until 16 hpi, however, at later time points the levels of protein phosphorylation detected in FAF1 knockdown cells were lower than those in scramble RAW264.7 cells. By contrast, FAF1-overexpressing RAW264.7 cells showed higher levels of phosphorylation at early time points than control cells (Fig 5, panel B). These results provide strong evidence that FAF1 activates the type I IFN signaling pathway. In addition, we examined phosphorylation of target proteins in FAF1 knockdown and FAF1-reconstituted MEFs after PR8-GFP infection (S8 Fig, panel A). The results showed that higher phosphorylation of these signaling proteins occurred at early time points in FAF1-reconstituted MEFs than in FAF1 knockdown MEFs. mRNA encoding IFN-related gene expressions were measured to determine whether type I IFN-related gene transcriptions were affected by FAF1 protein knockdown and reconstitution (Fig 5, panels C-D and S8 Fig, B-C). Lower mRNA expression levels were observed in FAF1 knockdown MEFs than WT MEFs (Fig 5, panel C), and significantly higher levels were noted in FAF1-reconstituted MEFs compared to FAF1 knockdown MEFs (S8 Fig, panel B). Furthermore, we examined the expression of mRNA encoding IFN-related genes in BMDMs and PBMCs isolated from FAF1^+/+^ mice and FAF1^gt/gt^ mice after infection with PR8-GFP or VSV-GFP (Fig 5, panel D and S8 Fig, panel C). Consistent with our previous findings, low levels of IFN-related gene transcription was observed in BMDMs and PBMCs of FAF1^gt/gt^ mice. To examine FAF1 expression levels in response to viral infection, the levels of FAF1 mRNA were measured in BMDMs, RAW264.7, THP-1, HEK293T, HeLa and A549 cells after PR8-GFP infection. As shown in S9 Fig, panel A, FAF1 mRNA expression levels were increased after viral infection, however, this increase varied according to cell type. Results from these experiments led us to postulate that FAF1 is a positive regulator of the type I IFN signaling pathway.

### FAF1 interacts with NLRX1

**Fig 6:**
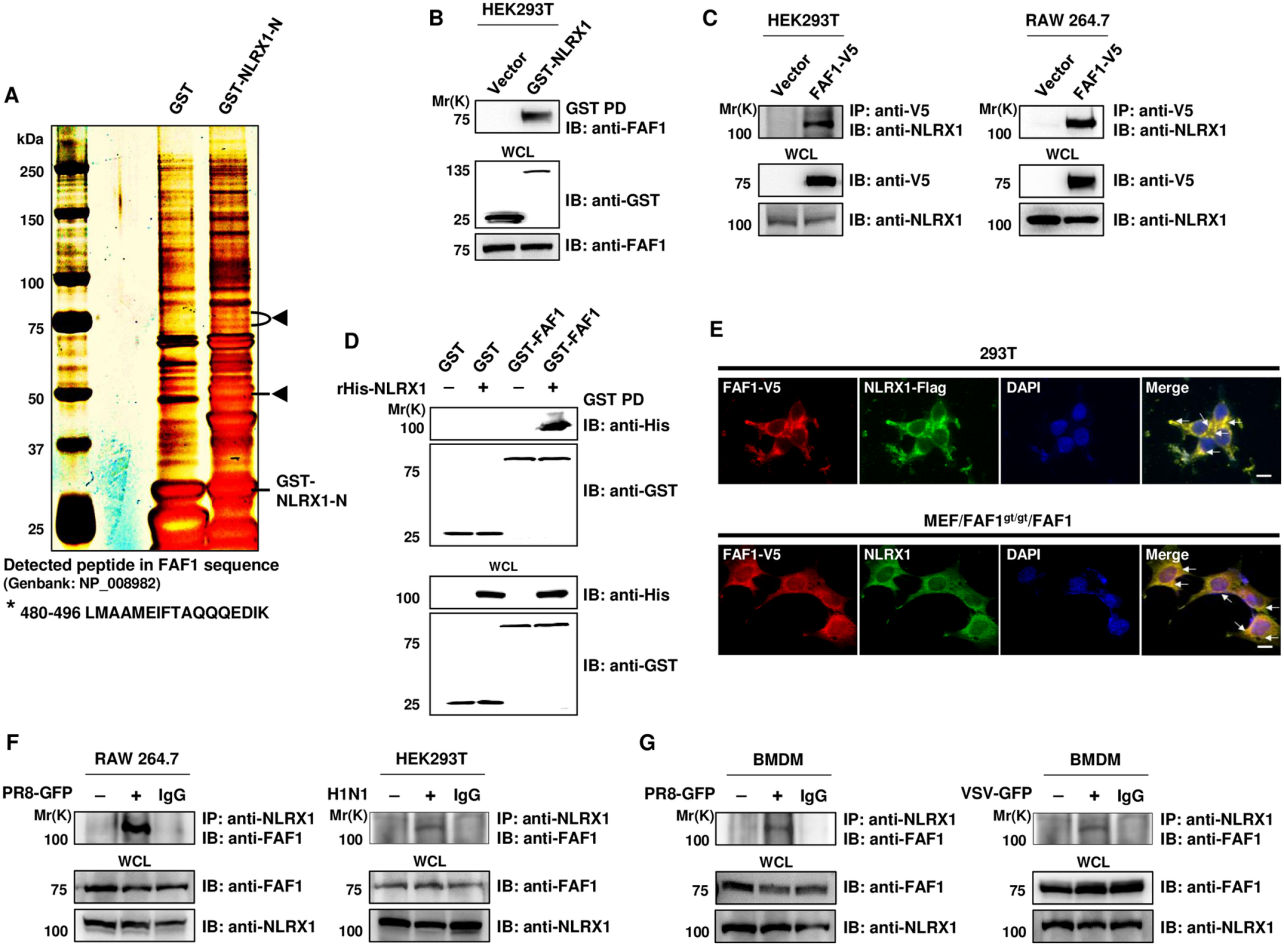
FAF1 interacts with NLRX1. (A) HEK293T cells were transfected with an empty GST vector (GST) or with the GST-NLRX1-N-terminal region (aa 1–225; GST-NLRX1-N). Proteins in the cell lysates were immunoprecipitated with GST beads and separated by 4–15% Nu-PAGE gels, followed by silver staining. Protein bands present exclusively in GST-NLRX1-N lane were excised from the gel and identified by mass spectrometry. The specific bands indicated by arrow heads were identified as FAF1. * indicates detected peptide corresponding to human FAF1 sequence. (B) WCL of HEK293T cells transfected with an empty GST vector or a GST-NLRX1 plasmid were subjected to a GST pull-down (GST PD) assay, followed by immunoblotting with anti-GST or anti-FAF1 antibodies. WCL were immunoblotted with anti-GST and anti-FAF1 antibodies. (C) WCL of HEK293T and RAW264.7 cells expressing FAF1-V5 proteins were immunoprecipitated with an anti-V5 antibody and immunoblotted with an anti-NLRX1 antibody. WCL were immunoblotted with anti-V5 and anti-NLRX1 antibodies. (D) *In vitro* binding assay, purified GST or GST-FAF1 protein immobilized on glutathione-conjugated Sepharose (GST) beads was incubated with the recombinant His tagged NLRX1 (rHis-NLRX1). After being pulled down, the bound proteins were subjected to immunoblotting with anti-GST and anti-His antibodies. WCL were immunoblotted with anti-GST and anti-His antibodies. (E) HEK293T cells transfected with FAF1-V5 and NLRX1-Flag plasmids were stained using anti-V5 and anti-Flag antibodies. FAF1-reconstituted MEFs (MEF/FAF1^gt/gt^/FAF1) were stained with anti-V5 and anti-NLRX1 antibodies. Secondary antibodies were labeled to identify localization of FAF1 (red; TRITC) and NLRX1 (green; FITC). Nuclei were stained using DAPI (blue). Yellow represents co-localization and marked in arrows. Bar, 10 μm. (F and G) RAW264.7, HEK293T and BMDMs cells were either mock-infected or infected with PR8-GFP (RAW264.7; MOI = 3, BMDMs; MOI = 3), H1N1 (HEK293T; MOI = 2) and VSV-GFP (BMDMs; MOI = 3MOI), and WCL were subjected to immunoprecipitation with an anti-NLRX1 antibody and control IgG, followed by immunoblot analysis with an anti-FAF1 antibody. WCL were immunoblotted with anti-FAF1 and anti-NLRX1 antibodies.

Previous studies in our laboratory focused on identifying binding partners for NLRX1. A large scale pull-down assay using HEK293T cells overexpressing the GST-tagged N-terminal domain (amino acids (aa) 1–225) of NLRX1 followed by mass spectrometry analysis identified that FAF1 is a binding candidate for NLRX1 (Fig 6, panel A). Immunoprecipitation of GST-tagged NLRX1 followed by immunoblotting with an anti-FAF1 antibody showed that NLRX1 interacted with FAF1 (Fig 6, panel B). Additionally, V5-tagged FAF1 was pull-down from HEK293T and RAW264.7 cell lysates, and NLRX1 was visualized by immunoblotting with an NLRX1 antibody (Fig 6, panel C). To further confirm whether FAF1 directly interacts with NLRX1, *in vitro* binding assay was performed using purified GST tagged FAF1 (Fig 6, panel D). Incubation of GST tagged FAF1 with recombinant His tagged NLRX1 followed by immunoblotting with anti-His antibody showed the thick band corresponding to the NLRX1 protein size, which indicates direct binding between FAF1 and NLRX1. Moreover, as shown in Fig 6, panel E, confocal microscopic visualization of overexpressed V5-tagged FAF1 and Flag-tagged NLRX1 in HEK293T cells or overexpressed V5-tagged FAF1 and endogenous NLRX1 in FAF1-reconstituted MEFs showed overlapping of NLRX1 and FAF1 spots, confirming their co-localization. To examine endogenous protein binding upon virus infection, FAF1 was immunoprecipitated from PR8-GFP or H1N1-infected HEK293T or RAW264.7 cells using an anti-FAF1 antibody, followed by immunoblotting with an anti-NLRX1 antibody, and band corresponds to NLRX1 was detected (Fig 6, panel F). Similar results were obtained from BMDMs of FAF1^+/+^ mice after infection of PR8-GFP or VSV-GFP (Fig 6, panel G). These results suggested that FAF1 interacts with NLRX1.

### FAF1 inhibits binding between MAVS and NLRX1

**Fig 7:**
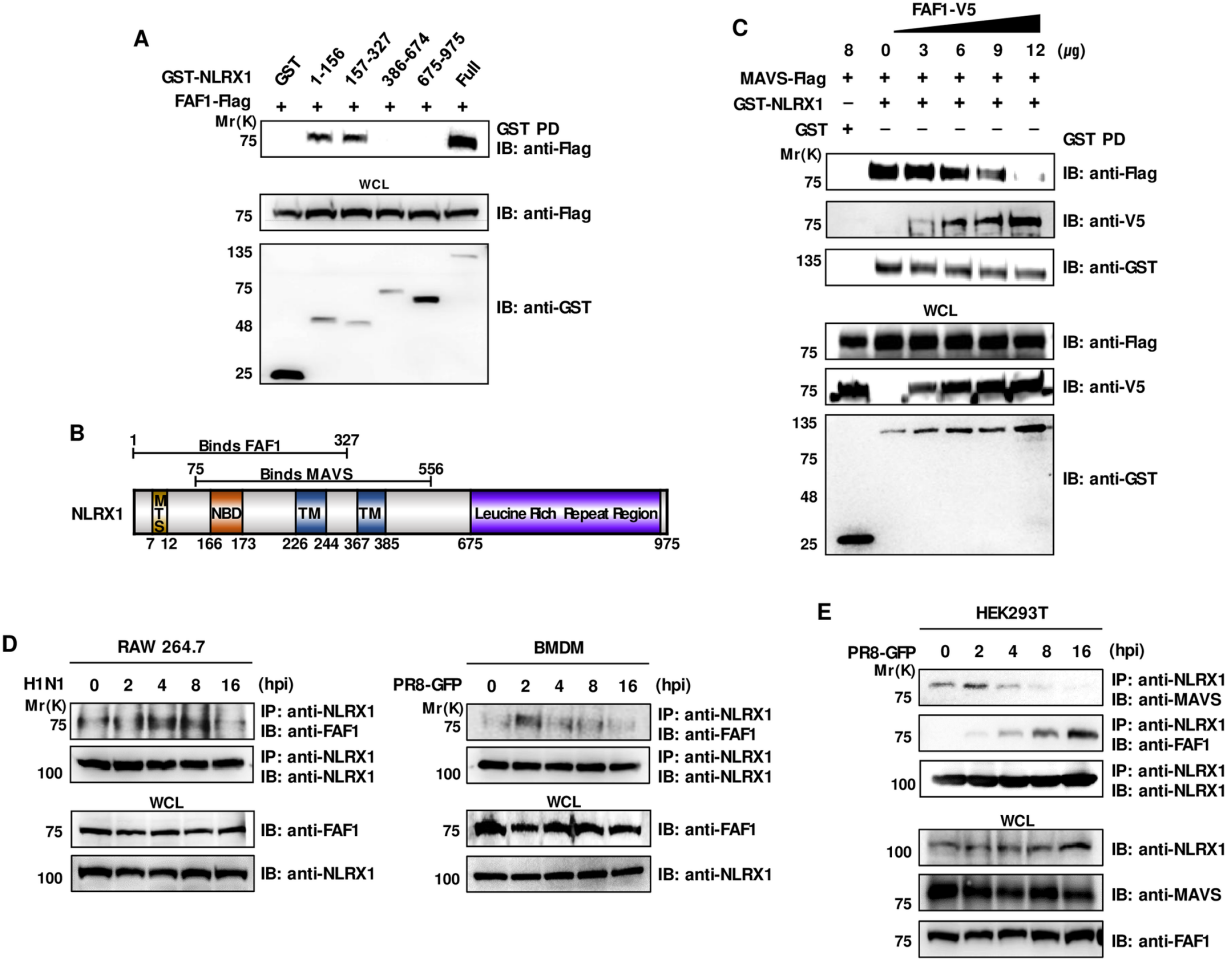
FAF1 inhibits the interaction between MAVS and NLRX1 after virus infection. (A) HEK293T cells were transfected with the indicated GST-NLRX1 constructs (aa 1–156, 157-.327, 386–674, 675–975) and FAF1-Flag. GST pull-down (GST PD) was conducted followed by immunoblot analysis with an anti-Flag antibody. WCL were immunoblotted with anti-Flag and anti-GST antibodies. (B) Structure of NLRX1. The carton schematically indicates the positions of the mitochondrial targeting sequences (MTS, aa 7–12) nucleotide-binding domain (NBD, aa 166–173), transmembrane domain (TM, aa 226–244 and 367–385) and leucin-rich-repeat (LRR, aa 675–975) containing family member (known as NLR). FAF1 and MAVS bind to aa 1–327 and aa 75–556 region of NLRX1, respectively. (C) HEK293T cells were co-transfected with different doses of a FAF1-V5 plasmid and MAVS-Flag and GST-NLRX1 plasmids. NLRX1-binding proteins were detected by immunoprecipitation of NLRX1 by GST pull-down (GST PD), followed by immunoblot analysis with anti-FLAG, anti-V5, and anti-GST antibodies. WCL were immunoblotted with anti-V5, anti-Flag and anti-GST antibodies. (D) RAW264.7 and BMDM cells were infected with H1N1 (MOI = 2) and PR8-GFP (MOI = 3), respectively. Cells were harvested at the indicated time points and NLRX1 protein was immunoprecipitated with an anti-NLRX1, followed by immunoblot analysis with anti-FAF1 and anti-NLRX1 antibodies. WCL were immunoblotted with anti-FAF1 and anti-NLRX1 antibodies. (E) HEK293T cells were infected with PR8-GFP (MOI = 3) for the indicated time points. Infected cells were harvested and subjected to immunoprecipitation with an anti-NLRX1 antibody. Immunoprecipitates were then immunoblotted with anti-MAVS, anti-FAF1 and anti-NLRX1 antibodies. Expression of NLRX1, MAVS, and FAF1 in the WCL was confirmed using anti-NLRX1, anti-MAVS, and anti-FAF1 antibodies.

To further elucidate the interaction between FAF1 and NLRX1, the NLRX1 domains responsible for the interaction with FAF1 were analyzed using GST-labeled NLRX1 domain constructs (Fig 7, panel A). This experiment revealed that amino acids (aa) 1–327 of NLRX1 are required for the interaction with FAF1. Moreover, we found that the FAF1 binding site within NLRX1 overlapped with the binding site for MAVS (aa 75–556) (Fig 7, panel B). For further confirmation, another two NLRX1 fragments were constructed (aa 556–975 and 75–975) to check whether FAF1 can bind with MAVS binding region of NLRX1 (S10 Fig, panel A). FAF1 bound to aa 75–975 of NLRX1, however, did not bind to aa 556–975 of NLRX1. This indicates that FAF1 binds with the aa 75–556 region of NLRX1 which binds with MAVS. Based on these findings, we hypothesized that FAF1 and MAVS compete for binding to NLRX1. To test this, a competition assay was performed by transfecting HEK293T cells with V5-tagged FAF1 in a dose-dependent manner (Fig 7, panel C). The results confirmed reduced binding between MAVS and NLRX1, meanwhile increased binding between FAF1 and NLRX1. Thus, FAF1 inhibits the interaction between NLRX1 and MAVS by binding to NLRX1 competitively. To investigate time-dependent changes in the interaction between FAF1 and NLRX1 after virus infection, RAW264.7 cells and BMDMs were infected with H1N1 or PR8-GFP and then harvested at different time intervals. Immunoprecipitation using an anti-NLRX1 antibody, followed by immunoblotting with an anti-FAF1 antibody, revealed that FAF1 bound to NLRX1 in RAW264.7 cells and BMDMs at early time points (4 and 2 hpi, respectively) (Fig 7, panel D). Additionally, the interaction between NLRX1 and FAF1 or NLRX1 and MAVS were examined in infected cells (Fig 7, panel E). The interaction between NLRX1 and MAVS in non-infected cells was markedly reduced after viral infection in a time-dependent manner, importantly, there was a corresponding increase in FAF1-NLRX1 binding. These results suggest that FAF1 interacts with NLRX1 and inhibits binding between MAVS and NLRX1. For further confirmation of this mechanism, we checked whether knockdown of NLRX1 abolishes the antiviral effect of FAF1 on type I IFN signaling. FAF1 and NLRX1 was knockdown in HEK293T cells using FAF1 and NLRX1 specific siRNA (S11 Fig, panel A), and then VSV-GFP was infected to the cells. First, we confirmed antiviral effect of FAF1 in HEK293T cells, as shown in S11 Fig, panel B-C. In knockdown of NLRX1, increased cytokine secretion and reduced VSV-GFP replication were observed. Interestingly, knockdown and overexpression of FAF1 had no effect to virus replication and cytokine secretion levels in NLRX1 knockdown condition. These results correlated with our proposed mechanism that FAF1 regulates NLRX1 mediated type I IFN signaling upon virus infection by interacting with NLRX1. Indeed, FAF1 competes with MAVS for binding to NLRX1, leading to disassociation of NLRX1 from MAVS, MAVS is then free to interact with RIG-I and initiate type I IFN signaling.

## Discussion

MAVS is the key adaptor protein for RLR-mediated signaling [15,39]. The RLR signaling pathway is initiated by recognition of distinct species of viral RNA by RIG-I or MDA5, and activated RLRs bind MAVS via CARD-mediated interactions [19]. MAVS then recruits downstream signaling molecules and, eventually, induces production of type I IFNs and proinflammatory cytokines [20,40]. Although activation of MAVS plays a role in inducing type I IFN to limit virus spread, it must be tightly modulated to prevent excessive cellular immune responses that may have a detrimental effect on the host [28,41]. After viral infection, MAVS is regulated negatively or positively by different mechanisms, including mitochondrial dynamics [42,43], post-translational modifications [44,45], or protein-protein interactions [35,46,47]. With respect to protein-protein interactions, the first protein to be identified as a negative regulator of MAVS was the nucleotide-binding domain and leucine-rich repeat containing family member, NLRX1 [35–38]. Although conflicting results have been reported with regard to the function of NLRX1 as a negative regulator of RLR-mediated antiviral signaling [48–50], it is believed that NLRX1 associates with MAVS on the mitochondrial membrane to inhibit antiviral signaling by interrupting virus-induced RLR-MAVS interactions [35–37,51]. However, the mechanism that regulates NLRX1 during virus infection remains poorly characterized.

FAF1, a member of the ubiquitin regulatory X (UBX) family, potentially interacts with diverse proteins and functions as a negative and/or positive regulator in variety of biological possesses, including apoptosis [1,3], tumor growth [2,4–7], protein degradation [2,6,52] and chaperone activity [53]. Here, we provide several lines of evidence showing that FAF1 is a positive regulator that modulates the type I interferon signaling pathway in response to RNA virus infection. First, FAF1^gt/gt^ mice were more susceptible to infection by VSV as they were permissive to high rates of virus replication and mounted weak antiviral immune responses. Second, knockdown of endogenous FAF1 in immune cells or MEFs from FAF1^gt/gt^ mice reduced RNA virus-induced IFN-β and proinflammatory cytokines production and increased viral replication. Third, overexpression of FAF1 in immune cells or MEFs promoted RLR-mediated antiviral response against RNA virus infection but not DNA virus infection. Fourth, FAF1 interacts with NLRX1 in response to RNA virus infection or RLR stimulation, and aa 1–327 of NLRX1 are responsible for the interaction between FAF1 and NLRX1. Finally, FAF1 interacts with NLRX1 at the early time points after RNA virus infection; this interaction inhibits binding of MAVS to NLRX1, which in turn switches on RIG-I mediated antiviral immune responses. Taken together, these findings indicate that FAF1 is a crucial regulator that induces the antiviral innate immune responses against RNA virus infection.

Recently, Song et.al., reported that FAF1 negatively regulates virus-induced IFN-β signaling and the antiviral response by inhibiting the translocation of active phosphorylated IRF3 from the cytosol to the nucleus [54]. However, this result contradicts that presented herein, and we clearly demonstrated that FAF1 acts as a positive regulator of the type I IFN signaling pathway during RNA virus infection. In particular, we identified the physiological role of FAF1 in innate immune responses against viral infection in FAF1^+/+^ and FAF1^gt/gt^ mice. FAF1^gt/gt^ mice were more susceptible to infection with VSV than FAF1^+/+^ mice, resulting high mortality in FAF1^gt/gt^ mice due to a high viral load in the organs. After virus infection or Poly (I:C) stimulation, FAF1^gt/gt^ mice showed lower levels of cytokine production (IL-6 and IFN-β) than FAF1^+/+^ mice, which strongly supporting an impaired antiviral immune response, especially with respect to type I IFN signaling.

We also evaluated antiviral responses in several cell types. BMDMs, BMDCs, and PBMCs isolated from FAF1^gt/gt^ mice, as well as FAF1 knockdown RAW264.7 cells and MEFs showed higher viral replication and lower IL-6 and IFN-β production, suggesting reduced antiviral and inflammatory responses due to suppression of FAF1. However, reconstitution of FAF1 in FAF1 knockdown MEFs restored the antiviral function of FAF1 by recovering production of these cytokines. Consistent with this, overexpression of FAF1 in RAW264.7 cells resulted enhanced antiviral responses. These results strongly support the involvement of FAF1 as a positive regulator of RNA virus-mediated type I IFN signaling. Here, we also examined the phosphorylation of IRF3 (the main integral component of the type I IFN response), p65 (a subunit of NF-κB), STAT1, p38, and TBK1 after the induction of IFN responses by PR8-GFP. Together with this, significantly decreased type I IFN, ISGs, and antiviral mRNA transcript levels were observed in BMDMs and PBMCs of FAF1^gt/gt^ mice compared with those in FAF1^+/+^ mice after infection with VSV and PR8.

Interestingly, our large scale co-immunoprecipitation data demonstrated that FAF1 is one of the binding partners for NLRX1 (Fig 6, panel A), which negatively regulates type I IFN signaling by modulating the interaction between RIG-I and MAVS [35–38]. We confirmed that FAF1 binds to and co-localizes with NLRX1. Moreover, we performed domain studies to better understand the mechanism underlying the interaction between FAF1 and NLRX1, and to identify which domain of NLRX1 binds to FAF1. The results showed that the MAVS binding site within NLRX1 (aa 75–556) [35] overlaps with the binding site for FAF1 (aa 1–327). Hence, we postulate that the mechanism by which FAF1 positively regulates IFN signaling probably operates through FAF1-mediated disassociation of NLRX1 from MAVS. This supports our time course binding studies, which showed that FAF1 binds to NLRX1 at early time points (2 and/or 4 hpi) after virus infection. We also examined the time course binding of NLRX1 and MAVS and compared it with that of NLRX1 and FAF1 after the virus infection. Similar to previous studies showing constitutive interaction between RIG-I and MAVS in NLRX1-deficient cells [38], we found that NLRX1 bound to MAVS in the absence of virus infection. This suggests that under normal conditions NLRX1 blocks the interaction between MAVS and RIG-I. However, after virus infection, FAF1 appears to displace MAVS from NLRX1 by competitive binding to NLRX1. Thus, we postulate that FAF1 stimulates type I IFN signaling pathway by sequestering NLRX1 from the RIG-I-MAVS-mediated pathway. Furthermore, we confirmed that knockdown of NLRX1 abolishes the FAF1 mediated effects on type I IFN signaling, which supports our proposed mechanism. Nevertheless, several controversial reports are related with NLRX1 [48–50], indicating more complicate mechanisms might involve on the role of NLRX1 on MAVS-dependent antiviral responses that unexplored yet, and FAF1 could be one of the key participant molecule in this mechanism which needs to be elucidated in future.

Recently, Guo et al. demonstrated that NLRX1 is a negative regulator of host innate immune responses to DNA viruses by sequestering the DNA-sensing adaptor, STING, from TANK-binding kinase 1 (TBK1) [55]. However, in this study, we found no differences in cytokine secretion levels or viral replication levels after HSV infection, suggesting that FAF1 may not modulate type I IFN production via STING-mediated sequestration of NLRX1 upon DNA virus infection. Hence, the present data indicate that FAF1 targets NLRX1 to regulate type I IFN production upon infection with RNA virus only. Moreover, the upstream signaling molecule that activates FAF1 after RNA virus infection and the reason that FAF1 only regulates NLRX1 upon RNA virus invasion remains unclear.

In summary, we showed that FAF1^gt/gt^ mice are highly susceptible to RNA virus infection and show defective innate immune responses both *in vitro* and in vivo. Upon RNA virus infection, FAF1 binds competitively to NLRX1, thereby preventing it from binding to MAVS; this frees MAVS to interact with RIG-I and switch on the antiviral signaling cascade. These results suggest a plausible and novel mechanism by which FAF1 positively regulates type I IFN signaling and increases our understanding of the molecules that control type I IFN signaling and antiviral immune responses.

## Methods

### Ethics statement

All animal experiments were managed in strict accordance with the Guide for the Care and Use of Laboratory Animals (National Research Council, 2011) and performed in BSL-2 and BSL-3 laboratory facilities with the approval of the Institutional Animal Care and Use Committee of Bioleaders Corporation (Reference number BLS-ABSL-14-009).

### *In vivo* mouse experiments

C57BL/6 FAF1^+/+^ and FAF1^gt/gt^ mice were kindly provided by Dr. Eunhee Kim (Department of Biology, Chungnam National University, Korea) [56]. Mice (6–7 weeks of age) were infected with vesicular stomatitis virus (VSV) Indiana strain (VSV-Indiana; 2 × 10^8^ pfu (plaque forming unit) per mouse) or green fluorescent protein (GFP)-tagged VSV (VSV-GFP; 2 × 10^8^ pfu per mouse) via tail vein injection. Mice infected with VSV-Indiana were observed daily to measure the mortality until 12 dpi. Organs and sera of mice infected with VSV-Indiana or VSV-GFP were collected at indicated time points to measure virus titers by plaque assays and qRT-PCR as described below, and levels of mouse IFN-β (PBL interferon source) and mouse IL-6 (BD biosciences) were measured by ELISA. Poly (I:C) (Invivogen; 200 mg per mouse) was injected intravenously via tail vein, and the sera were collected to measure mouse IFN-β and IL-6 levels by ELISA at indicated time points. Peripheral blood mononuclear cells (PBMCs) were isolated from whole pheripheral blood of FAF1^+/+^ and FAF1^gt/gt^ mice infected with VSV-GFP (4 × 10^8^ pfu per mouse) via tail-vein injection at 24 hpi as described below. Total RNAs from PBMCs were extracted and were used for qRT-PCR analysis as described below.

### Immune cells isolation from FAF1^+/+^ and FAF1^gt/gt^ mice

The femurs and tibias were isolated from euthanized C57BL/6 mice (4–6 weeks of age) aseptically. After removing muscles, the bones were flushed with Dulbecco’s Modified Eagle’s medium (DMEM, Gibco) using syringe (26G × ½ needle) to extrude bone marrow at least 3 times. After centrifugation of bone marrow, pellet was re-suspended with 1.0 ml of ammonium-chloride-potassium (ACK) lysis buffer (Gibco) to lyse the red blood cells, and supernatant was aspirated from the white cell pellet after centrifugation. Cells were cultured with DMEM supplemented with 10% heat-inactivated fetal bovine serum (FBS, Gibco) and 1% antibiotic-antimycotic (Gibco) (10% FBS DMEM) and 10 ng/ml granulocyte-macrophage colony-stimulating factor (GM-CSF) for 5 days to obtain Bone Marrow-Derived Macrophages (BMDMs). Additionally, cells were cultured for 6 days by adding 100 ng/ml IL-4 (Invivogen) to the above media to prepare Bone Marrow-Derived Dendritic Cells (BMDCs). Whole peripheral blood obtained from mice was diluted with roswell park memorial institute (RPMI) 1640 medium (Gibco) and PBMCs were isolated by Histopaue-1077 (Sigma). Isolated PBMCs were washed 3 times and cultured in 10% FBS and 1% antibiotic-antimycotic included RPMI1640 medium (10% FBS RPMI).

### Cell culture

FAF1 knock-down murine embryonic fibroblasts (MEFs) provided by Dr. Eunhee Kim (Department of Biology, Chungnam National University, Korea) [56], mouse leukaemic monocyte macrophage (RAW264.7; ATCC TIB-71), human embryonic kidney 293 (HEK293T; ATCC CRL-11268), human epithelial cervix adenocarcinoma (HeLa; ATCC CCL-2) and adenocarcinomic human alveolar basal epithelial (A549; ATCC CCL-185) cell line were grown and maintained in 10% FBS DMEM at 37°C and 5% CO_2_. Human acute monocytic leukemia (THP-1; ATCC TIB-202) cell line was grown and maintained 10% FBS RPMI.

### Plasmids

FAF1 tagged with V5 expression plasmid (pIRES-FAF1-V5) was constructed by inserting the FAF1 complete ORF which was amplified from pFLAG-CMV-2/hFAF1 plasmid [6] to the pIRES-V5 vector between *Afl*II and *EcoR*I site. NLRX1 inserted to pIRES plasmid was kindly donated by Dr. Jae U. Jung (Department of Molecular Microbiology and Immunology, University of Southern California, USA), and GST tagged NLRX1 full (aa 975) and 6 fragments (aa 1–156, 157-.327, 386–674, 675–975, 556–975 and 75–975) were constructed by cloning into the pEBG vector between *BamHI* and *NotI* site.

### Generation of stable cell lines

For stable overexpressing cell line preparation, pIRES-V5 vector or pIRES-FAF1-V5 was transfected to RAW264.7 and HEK293T with Lipofectamine 2000 (Invitrogen) according to manufacturer’s protocol. Cells stably expressing pIRES-V5 and pIRES-FAF1-V5 were selected with 2 μg/ml puromycin (Gibco) containing 10% FBS DMEM for 2 weeks.

### Virus infection and stimulant transfection

VSV-GFP, GFP tagged Herpes Simplex virus 1 (HSV-GFP) and Adenovirus (Adeno-GFP) were propagated in *Ceropithecus aethiops* epithelial kidney (Vero; ATCC CCL-81) cells. GFP tagged H1N1 influenza virus (A/PR8/8/34; PR8-GFP) and Newcastle disease virus (NDV-GFP) were propagated in embryonated chicken eggs. Culture medium was replaced by DMEM supplemented with 1% FBS right before virus infection, and the viruses were added into the medium with indicated MOI. After 2 hr incubation, extracellular virus was removed and replace with 10% FBS DMEM or RPMI. Poly (I:C) was transfected with Lipofectamine 2000 into MEFs or treated to RAW264.7 cells. 5’- triphosphate double-stranded RNA (5’ppp-dsRNA, Invivogen) was transfected into both cell lines with Lipofectamine RNAiMAX (Invitrogen). Imiquimod (Invitrogen) and ODN2395 (Invitrogen) were treated to RAW264.7 cells.

### Lentiviral shRNA

Oligonucleotide sequences of FAF1-specific shRNA cloned into the pGIPZ lentiviral vector expressing GFP was purchased at Open Biosystems. (http://www.openbiosystems.com). Lentiviruses were produced using transient transfection of packaging plasmids (psPAX2 and pMD2.VSV-G purchased from Addgene) into HEK293T cells using Lipofectamine 2000. Media supernatant containing the virus particles were collected after 72 hr, filtered (0.45 μm filter, Millipore) and infected to the RAW264.7 cells with 8μg/ml polybrene (Sigma). Culture medium was replaced after the transduction process (after 12hr) with fresh puromycine-containing medium every 2 days until resistant colonies could identified. Similarly, control cells were prepared by infecting lentivirus which was produced with pGIPZ lentiviral vector expressing GFP.

### siRNA experiment

To knockdown the FAF1 or NLRX1 gene expression, siRNA oligonucleotide duplexes for targeting mouse FAF1 (si-mFAF1-S 5’-UGUUUCCCUGGGACCAUCU-3’ and si-mFAF1-AS 5’-AGAUGGUCCCAGGGAAACA-3’), human FAF1 (si-hFAF1-S 5’-CAGUAGAUGAGUUAAUGAU-3’ and si-hFAF1-AS 5’-AUCAUUAACUCAUCUACUG-3’) or human NLRX1 (si-hNLRX1-S 5’-GAGGAGGACUACUACAACGAU-3’ and si-hNLRX1-AS 5’- AUCGUUGUAGUAGUCCUCCUC-3’) was transfected to cells (si-mFAF1; RAW264.7 cells, si-hFAF1; THP-1 and HEK293T cells and si-hNLRX1; HEK293T cells) using Lipofectamine RNAiMAX according to the manufactures protocol.

### Virus titer determination

To measure virus titer, supernatant of homogenized organs (VSV-Indiana), cells (VSV-GFP, NDV-GFP and HSV-GFP) or freezed-thawed cells (PR8-GFP and Adeno-GFP) which collected at indicated time points were serially 10-fold diluted and inoculated to Vero cells in 1% FBS containing media. After incubation for 2 hr at 37°C, cells were overlaid with DMEM containing 1% agarose (Sigma). Cultures were incubated at 37°C, 5% CO_2_ for 48 hr, plaques were visualized with crystal violet. Virus titer was calculated using the number of plaques and the dilution factor. GFP expression levels were measured using a fluorescence modulator (GloMax-Multi detection system; Promega) to digitize.

### ELISA

ELISA was used to detect the production of pro-inflammatory cytokines and type I interferon from cells. After infection, treatment and transfection of stimulants, cell supernatant was collected and analyzed cytokine production levels. Mouse IFN-α (PBL interferon source), mouse IFN-β (PBL interferon source), mouse IL-6 (BD biosciences) and mouse TNF-α (BD biosciences), human IFN-β (PBL interferon source) and human IL-6 (BD biosciences) were used for analysis according to manufacturer’s protocol.

### GST pull-down and immunoprecipitation

At 48 hr post-transfection of indicated plasmids, cells were harvested and lysed with radio-immunoprecipitation assay (RIPA) lysis buffer (50 mM Tris-HCl, 150 mM NaCl, 0.5% sodium deoxycholate, 1% IGEPAL, 1 mM NaF, 1 mM Na_3_VO_4_) supplemented with protease inhibitor cocktail and phosphatase inhibitor cocktail (Sigma) and sonicated to prepare the whole cell lysate (WCL). WCL were precleared with Sepherose 6B (GE Healthcare Life Science) at 4°C at least for 2 hr. Precleared lysates were incubated with 50% slurry of glutathione-conjugated Sepharose (GST) beads (Amersham Biosciences) for GST pull-down, and for immunoprecipitation of anti-V5 and NLRX1, lysates were incubated with anti-V5 or NLRX1 antibody (1.0 μg/ml) for 12 hr and then protein A/G plus agarose beads (Santacruz) were added. Immunoprecipitates were collected by centrifugation, washed with lysis buffer in different washing conditions.

Additionally, WCL in control, FAF1 knockdown and overexpressing RAW264.7 and FAF1 knockdown and reconstituted MEFs infected with PR8-GFP during indicated time points were subjected to immunoblotting to analyze protein phosphorylation levels using respective antibodies.

### Immunoblot analysis

For all the immunoblot analysis, samples were separated by SDS-PAGE and transferred onto a PVDF membrane (Bio-rad) using Trans-Blot semi dry transfer cell (Bio-rad). Membranes were blocked for 1 hr in tris-buffered saline containing 0.05% tween 20 (TBST) containing 5% bovine-serum albumin (BSA). After overnight-incubation at 4°C with antibodies, membranes were washed with TBST. Membranes were incubated at room temperature with 1:3000 dilutions of horseradish peroxidase-conjugated secondary antibodies. Membranes were developed with western blotting detection reagents (GE healthcare, ECL select Western Blotting Detection Reagent).

### Antibodies

The antibodies used in this study were as follows: anti-GST (Santacuze, #SC-138), anti-V5 (Invitrogen, #46–0705), or anti-IRF3 (Abcam, #ab25950), anti-phospho-IRF3 (Ser 396) (Cell signaling, #4947), anti-NF-κB p65 (Cell signaling, #4764), anti-phospho-NF-κB p65 (Ser536) (Cell signaling, #3031), anti-STAT1 (Cell signaling, #9175), anti-phospho-STAT1 (Cell signaling, #9167), anti-phospho-p38 (Cell signaling #9216), phospho-TBK1 (Cell signaling #5483), anti-NLRX1 (Proteintech, #17215-1-AP) and anti-His (Santacuze, #SC-1803) antibodies. The anti-FAF1 monoclonal antibody was provided by Dr. Eun-hee Kim (Department of Biology, Chungnam National University, Korea). The anti-interferon-α/β receptor (IFNAR) (25 μg/ml; Leinco Technologies) was pre-incubated in RAW264.7 cells and MEFs for 1 hr before VSV-GFP infection to block IFNAR.

### mRNA expression analysis by qRT-PCR

Total RNA was isolated from cells and tissues from the organs using RNeasy Mini Kit (Qiagen) and cDNA synthesis was performed using ReverTra Ace kit (TOYOBO). cDNAs were then quantified with gene specific primer pairs using QuantiTect SYBR Green PCR kit (Qiagen) on a Rotor-Gene Q (Qiagen) and relative expression of mRNA was normalized to GAPDH mRNA expression using delta-delta CT method. Gene specific primer pairs were referred in Table 1.

**Table 1:** Primer sets used to confirm mRNA expression.

Genes	Primers	
	Forward	Reverse
VSV	5'-TGATACAGTACAATTATTTTGGGAC-3'	5'-GAGACTTTCTGTTACGGGATCTGG-3'
mGAPDH	5'-TGACCACAGTCCATGCCAT-3'	5'-GACGGACACATTGGGGGTAG-3'
mIFN-β	5'-TCCAAGAAAGGACGAACATTCG-3'	5'-TGCGGACATCTCCCAACGTCAA-3'
mIFN-α	5'-CTTGAAGGACAGACATGACTTTGGA-3'	5'-GGATGGTTTCAGCCTTTTGGA-3'
mPKR	5'-GCCAGATGCACGGAGTAGCC-3'	5'-GAAAACTTGGCCAAATCCACC-3'
mOAS	5'-GAGGCGGTTGGCTGAAGAGG-3'	5'-GAGGAAGGCTGGCTGTGATTGG-3'
mOAS-1β	5'-TTGATGTGCTGCCAGCCTAT-3'	5'-TGAGGCGCTTCAGCTTGGTT-3'
mMX-1	5'-ACAAGCACAGGAAACCGTATCAG-3'	5'-AGGCAGTTTGGACCATCTTAGTG-3'
mISG15	5'-CAATGGCCTGGGACCTAAA-3'	5'-CTTCTTCAGTTCTGACACCGTCAT-3'
mISG20	5'-AGAGATCACGGACTACAGAA-3'	5'-TCTGTGGACGTGTCATAGAT-3'
mISG56	5'-AGAGAACAGCTACCACCTTT-3'	5'-TGGACCTGCTCTGAGATTCT-3'
mADAR1	5'-CCAAAGACACTTCCTCTC-3'	5'-CAGTGTGGTGGTTGTACT-3'
mPML	5'-CCTGCGCTGACTGACATCTACT-3'	5'-TGCAACACAGAGGCTGGC-3'
mGBP1	5'-AAAAACTTCGGGGACAGCTT-3'	5'-CTGAGTCACCTCATAAGCCAAA-3'
mIL-6	5'-GACAACTTTGGCATTGTGG-3'	5'-ATGCAGGGATGATGTTCTG-3'
mFAF1	5'-GGTGACTGCCATCCTGTATTTT-3'	5'-TGCTCTGTTGGTGTCCTTTG-3'
hβ-actin	5'-GGGGATCCGGCGACGAGGCCCAGAGCAAG-3'	5'-TTCAACACCCCAGCCATGTACGGATCCCC-3'
hFAF1	5'-CTGCAGGAGTCATTAAATC-3'	5'-ATGG CAGGGATAAGAGAGCCC-3'

h: human

m: mouse

### Immunofluorescence and confocal analysis

The cells were seeded into collagen-coated chamber slides (LabTek, Nunc), 1 day prior to the experiments. Following day, the cultured cells were washed with phosphate buffer saline (PBS) and fixed with 4% paraformaldehyde for 20 min, then permeabilized through incubation for 20 min with 100% methanol at -20°C. The fixed cells were first incubated with 2% FBS diluted in PBS for 1 hr to block non-specific binding of antibodies. V5 and NLRX1 were detected through incubation with the primary antibodies (1:100 diluted in 2% BSA) for 12 hr at 4°C. After 3 times PBS containing 0.05% tween 20 (PBST) washing, the secondary antibodies (1:100 diluted in 2% BSA; Alexa 488 goat anti-rabbit IgG (Invitrogen), Cy3-conjugated donkey anti-mouse IgG (The Jackson Laboratory) were added and the cells were incubated for 1 hr at room temperature. Three times PBST washing followed by 10 min incubation with 1 μg/ml DAPI (Sigma-Aldrich) containing 0.01% RNase A, the nuclei were visualized, and then the slides were mounted with mounting solution (VECTOR) to check under fluorescence microscopy. Images were acquired from Nikon C2 Plus confocal microscope (Nikon) consisting of a Nikon Eclipse Ti inverted microscope with a confocal scanning system (Nikon) in conjunction with C-HGFIE precentered fiber illuminator (Nikon). FITC and TRITC fluorescence was detected using the 488 nm and 561 nm laser line of a Sapphire driver unit (Coherent), respectively, and DAPI fluorescence was detected using 405 nm laser line of a CUBE laser system (Coherent). The image data were analyzed using NIS-Elements microscope imaging software program (Nikon).

### Silver staining and mass spectrometry analysis

HEK293T cells were transfected with an empty GST vector (GST) or with the GST-NLRX1-N-terminal region containing vector (aa 1–225; GST-NLRX1-N). Cells were harvested after 48 hr and after cell lysis, proteins in the cell lysates were immunoprecipitated with GST beads and separated by 4–15% Nu-PAGE gels (Invitrogen), followed by silver staining [57]. Protein bands present exclusively in GST-NLRX1-N lane were excised from the gel and identified by mass spectrometry.

### *In vitro* binding assay

GST and GST tagged FAF1 (GST-FAF1) were expressed and purified using GST beads. The purified GST-FAF1 was incubated with recombinant His tagged NLRX1 (NovoPro) in binding buffer (50 mM Tris-HCl, 150 mM NaCl, 1% IGEPAL and protease inhibitors) at 4°C for 3 hr with gentle rocking. After centrifugation, collected beads were washed five times with binding buffer, and bound proteins were subjected to SDS-PAGE followed by immunoblotting with GST and His antibodies.

### Statistical analysis

Statistical analysis was performed using GraphPad Prism software version 6 for Windows (GraphPad Software). All the data were from at least of two independent experiments and data are shown as mean ± SEM. The means values of all the *in vitro* experiments were compared by Student’s t test. Log Rank test and Mann-Whitney test was subjected for in vivo survival data analysis. Comparisons between multiple time points were analyzed by one way analysis of variance (ANOVA). In all experiments, p values of less than 0.05 were considered statistically significant. *p<0.05, **p<0.01 and ***p<0.001

## Supporting information

S1 Fig Immune responses were decreased in FAF1^gt/gt^ mice upon VSV-Indiana infection and Poly (I:C) treatment. (A and C) The genotypes of the wild-type (FAF1^+/+^) and FAF1 knockdown (FAF1^gt/gt^) mice were conducted by generating PCR fragments from tail DNA (A) and from isolated organs (lung, liver, spleen, large intestine and small intestine) (B). PCR fragments were generated with the primers (C) of WT FAF1 allele from FAF1^+/+^ mice and trapped FAF1 allele from FAF1^gt/gt^ mice. Mouse GAPDH primers were used as an internal reference gene (positive control). (D) FAF1^+/+^ (n = 4) and FAF1^gt/gt^ (n = 4) mice whole organs (spleen, lung, liver and brain) were collected at 24 hpi of VSV-Indiana (2 × 10^8^ pfu/mouse) via tail-vein injection. The viral load in supernatants of homogenized organs were measured by qRT-PCR. Data represent mean ± SD. *P < 0.05 and **P < 0.01 as compared between the indicated groups (Student’s t test). (E) FAF1^+/+^ (n = 5) and FAF1^gt/gt^ (n = 5) mice were injected with Poly (I:C) (200 μg per mouse) via tail-vein injection. Sera were collected from the mice at indicated time points and IL-6 and IFN-β were measured by ELISA. Data represent mean ± SD. **P < 0.01 as compared between the indicated groups (Student’s t test).
(PDF)

S2 Fig BMDCs and PBMCs isolated from FAF1^gt/gt^ mice showed high virus replication and low cytokine (IL-6 and IFN-β) secretion against virus infection. (A and B) Wild-type BMDCs (BMDC/FAF1^+/+^) or FAF1 knockdown BMDCs (BMDC/FAF1^gt/gt^) were incubated with VSV-GFP (MOI = 2), PR8-GFP (MOI = 3), or Poly (I:C) (20 μg/ml). (C and D) Wild-type (PBMC/FAF1^+/+^) and FAF1 knockdown PBMCs (PBMC/FAF1^gt/gt^) were infected with VSV-GFP (MOI = 2). Virus titers were measured by plaque assay (A and C) and qRT-PCR (c). IL-6 and IFN-β levels were evaluated by ELISA (B and D). Data represent mean ± SD. *P < 0.05, **P < 0.01 and ***P < 0.001 as compared between the indicated groups (Student’s t test).
(PDF)

S3 Fig Knockdown of FAF1 inhibited the immune responses but restored after reconstitution of FAF1 in MEFs. (A) Confirmation of FAF1 protein levels in wild-type MEFs (MEF/FAF1^+/+^) and FAF1 knockdown MEFs (MEF/FAF1^gt/gt^) by immunoblot analysis. (B) GFP expression levels of MEF/FAF1^+/+^ and MEF/FAF1^gt/gt^ infected with NDV-GFP were visualized at 24 hpi, under fluorescence microscopy (200 × magnification), and quantified using a fluorescence modulator. Virus titers were determined by plaque assay. Data represent mean ± SD. **P < 0.01 as compared between the indicated groups (Student’s t test). (C) IL-6 and IFN-β levels in cell supernatants harvested from MEF/FAF1^+/+^ and MEF/FAF1^gt/gt^/FAF1 were measured by ELISA at 12 and 24 hpi of NDV-GFP. Data represent mean ± SD. **P < 0.01 and ***P < 0.001 as compared between the indicated groups (Student’s t test). (D) Reconstitution of FAF1 was evaluated by analyzing the levels of FAF1-V5 and β-actin in MEF/FAF1^gt/gt^ and FAF1 reconstituted MEF/FAF1^gt/gt^ (MEF/FAF1^gt/gt^/FAF1) by immunoblot analysis. β-actin was used to confirm equal protein loading. (E and F) MEF/FAF1^gt/gt^ and MEF/FAF1^gt/gt^/FAF1 were infected with VSV-GFP (MOI = 0.5), PR8-GFP (MOI = 1) or NDV-GFP (MOI = 1). GFP expression was visualized at 24 hpi, under fluorescence microscopy (200 × magnification), and quantified using a fluorescence modulator. Virus titers were measured by plaque assay (E). Data represent mean ± SD. *P < 0.05, **P < 0.01 and ***P < 0.001 as compared between the indicated groups (Student’s t test). Levels of IL-6 and IFN-β in cell supernatants were assayed by ELISA at 12 and 24 hpi (F). Data represent mean ± SD. *P < 0.05, **P < 0.01 and ***P < 0.001 as compared between the indicated groups (Student’s t test). (G) MEF/FAF1^gt/gt^ and MEF/FAF1^gt/gt^/FAF1 were treated with Poly (I:C) (20 μg/ml) or 5’ppp-dsRNA (1 μg/ml), and levels of IL-6 and IFN-β in cell supernatants were assayed by ELISA after 12 or 24 hr of treatment. Data represent mean ± SD. *P < 0.05, **P < 0.01 and ***P < 0.001 as compared between the indicated groups (Student’s t test).
(PDF)

S4 Fig Knockdown of FAF1 negatively regulated type I IFN secretion against virus infection in RAW264.7 and THP-1 cells. (A) Confirmation of FAF1 protein levels in control RAW264.7 (RAW-Scramble), FAF1 shRNA knockdown RAW264.7 (RAW-shRNA-FAF1) and FAF1 siRNA knockdown RAW264.7 (RAW-siRNA-FAF1) cells by immunoblot analysis. β-actin was used to confirm equal protein loading. (B and C) RAW-Scramble and RAW-siRNA-FAF1 were infected with VSV-GFP (MOI = 1), and GFP expression was visualized under a fluorescence microscopy (200 × magnification) and quantified using a fluorescence modulator at 12 and 24 hpi. Virus titers were determined by plaque assay (B). Data represent mean ± SD. ***P < 0.001 as compared between the indicated groups (Student’s t test). IFN-β levels in cell supernatants were analyzed by ELISA (C). Data represent mean ± SD. *P < 0.05 and **P < 0.01 as compared between the indicated groups (Student’s t test). (D) Confirmation of FAF1 protein levels in control THP-1 (THP-1-Scramble) and FAF1 knockdown THP-1 (THP-1-siRNA-FAF1) by immunoblot analysis. (E and F) THP-1-Scramble and THP-1-siRNA-FAF1 were infected with VSV-GFP (MOI = 1), and GFP expression was visualized under a fluorescence microscopy (200 × magnification) and quantified using a fluorescence modulator at 12 and 24 hpi. Virus titers were determined by plaque assay (E). Data represent mean ± SD. *P < 0.05 and **P < 0.01 as compared between the indicated groups (Student’s t test). IFN-β levels in cell supernatants were analyzed by ELISA (F). Data represent mean ± SD. *P < 0.05 and **P < 0.01 as compared between the indicated groups (Student’s t test). (G) THP-1-Scramble and THP-1-siRNA-FAF1 were treated with Poly (I:C) (20 μg/ml), and levels of IFN-β in cell supernatants were assayed by ELISA. Data represent mean ± SD. **P < 0.01 as compared between the indicated groups (Student’s t test).
(PDF)

S5 Fig Overexpression of FAF1 positively regulated type I IFN secretion against virus infection in RAW264.7 cells. (A) Confirmation of FAF1 protein levels in control RAW264.7 (RAW-Control) and FAF1 overexpressing RAW264.7 (RAW-FAF1) by immunoblot analysis. Immunoblotting of β-actin was used to confirm equal loading. (B and C) RAW-Control and RAW-FAF1 were infected with NDV-GFP (MOI = 1), and GFP expression was visualized under a fluorescence microscopy (200 × magnification) and quantified using a fluorescence modulator at 24 hpi. Virus titers were determined by plaque assay (B). Data represent mean ± SD. *P < 0.05 and **P < 0.01 as compared between the indicated groups (Student’s t test). IL-6, IFN-α, and IFN-β levels in cell supernatants were analyzed by ELISA (C). Data represent mean ± SD. **P < 0.01 and ***P < 0.001 as compared between the indicated groups (Student’s t test). (D) RAW-Control and RAW-FAF1 were treated with Poly (I:C) (20 μg/ml) or 5’ppp-dsRNA (1 μg/ml), and levels of IL-6, IFN-α, and IFN-β in cell supernatants were assayed by ELISA. Data represent mean ± SD. *P < 0.05, **P < 0.01 and ***P < 0.001 as compared between the indicated groups (Student’s t test).
(PDF)

S6 Fig FAF1 has no role in antiviral activity upon DNA virus infection in RAW264.7 cells. (A) RAW264.7 cells were infected with lentivirus harboring scramble and FAF1 shRNA to prepare control RAW264.7 (RAW-Scramble) and FAF1 knockdown RAW264.7 (RAW-sh-FAF1), respectively. Cells were infected with Adenovirus (MOI = 4). After 24 hr, the virus titer was measured by plaque assay, and IL-6, IFN-α, and IFN-β levels in the supernatant were measured by ELISA at 12 and 24 hpi. Data are presented as the mean ± SEM. Data are representative of at least two independent experiments. (B) Stably expressing control (RAW-Control) and FAF1-overexpressing (RAW-FAF1) cells were infected with VSV-GFP (MOI = 1). At 24 hpi, GFP expression was visualized under a fluorescence microscopy (200 × magnification) and quantified using a fluorescence modulator. Virus titers were measured by plaque assay. Culture supernatants were collected at 12 h and 24 hpi, and IL-6 and IFN-β levels were measured by ELISA. Data are presented as the mean ± SEM. Data are representative of at least two independent experiments.
(PDF)

S7 Fig FAF1 regulate type I IFN signaling through RIG-I-MAVS pathway, not through TLR7 or TLR9. (A and B) Non-treated or anti-IFNAR antibody treated control RAW264.7 (RAW-Scramble) and FAF1 knockdown RAW264.7 (RAW-sh-FAF1) cells (A) or Wild-type MEFs (MEF/FAF1^+/+^) and FAF1 knockdown MEFs (MEF/FAF1^gt/gt^/FAF1) (B) were infected with VSV-GFP (MOI = 1 or 0.5, respectively). After 16 hr, GFP expression was visualized under a fluorescence microscopy (200 × magnification) and quantified using a fluorescence modulator. The virus titer was measured by plaque assay. Data are presented as the mean ± SEM. **P < 0.01 (Student’s t test). Data are representative of at least two independent experiments. (C) Control RAW264.7 (RAW-Scramble) and FAF1 knockdown RAW264.7 (RAW-sh-FAF1) cells were treated with imiquimod (2 μg/ml) or ODN2395 (2 μM). At 12 and 24 hpi, culture supernatants were collected, and IL-6 and IFN-β levels were measured by ELISA. Data are presented as the mean ± SEM. Data are representative of at least two independent experiments.
(PDF)

S8 Fig FAF1 enhances type I IFN signaling and expression of mRNA encoding IFN and IFN-related gene. (A) The amount of phosphorylated IRF3, p65, STAT1, p38, and TBK1 and total IRF3, p65, and STAT1 was examined by immunoblot analysis of cell extracts from FAF1 knockdown MEFs (MEF/FAF1^gt/gt^) and FAF1-reconstituted MEF/FAF1^gt/gt^ (MEF/FAF1^gt/gt^/FAF1) at the indicated times after PR8-GFP (MOI = 3) infection. β-actin was used to confirm equal protein loading. (B and C) MEF/FAF1^gt/gt^ and MEF/FAF1^gt/gt^/FAF1 (B) and PBMCs isolated from FAF1^+/+^ (PBMC/FAF1^+/+^) and FAF1^gt/gt^ (PBMC/FAF1^gt/gt^) mice (C) were infected with PR8-GFP (MOI = 1 and 3, respectively) for 12 hr. Total RNA was extracted from infected cells to determine the expression of mRNA encoding IFN-β, IFN-α, PKR, OAS, MX-1, ISG-15, ISG-20, ISG-56, ADAR1 and IL-6 for MEFs and IFN-β, PKR, OAS, OAS-1β, ISG-15, ISG-20 and ISG-56 for PBMCs was analyzed by qRT-PCR. Data represent mean ± SD.
(PDF)

S9 Fig FAF1 mRNA expression was increased in various cell types after PR8-GFP infection. (A) Different cell types were infected with PR8-GFP (BMDMs, MOI = 3, RAW264.7, MOI = 2; MEFs, MOI = 1; THP-1, MOI = 3; HEK293T, MOI = 1; HeLa, MOI = 2; and A549, MOI = 2). Total RNA was extracted from infected cells at indicated time points and FAF1 mRNA expression was analyzed by qRT-PCR. Data represent mean ± SD.
(PDF)

S10 Fig FAF1 binds to MAVS binding region of NLRX1. (A) HEK293T cells were transfected with the indicated GST-NLRX1 constructs (aa 556–975, 75–975 and 1–975) and FAF1-V5. GST pull-down (GST PD) was conducted followed by immunoblot analysis with anti-V5 and anti-GST antibodies. WCL were immunoblotted with anti-V5 and anti-GST antibodies.
(PDF)

S11 Fig Knockdown of NLRX1 abrogate FAF1 mediated antiviral effect of type I IFN in HEK293T cells. (A) Confirmation of FAF1 and NLRX1 protein levels in control (Scramble) and FAF1 (siRNA-FAF1) or NLRX1 (siRNA-NLRX1) siRNA knockdown HEK293T cells by immunoblot analysis. β-actin was used to confirm equal protein loading. (B) Control (293T-Control), FAF1 knockdown (293T-si-FAF1) NLRX1 knockdown (293T-si-NLRX1) and NLRX1/FAF1 knockdown (293T-si-NLRX1/si-FAF1) HEK293T cells were infected with VSV-GFP (MOI = 0.001), and GFP expression was visualized under a fluorescence microscopy (200 × magnification) and quantified using a fluorescence modulator at 12 hpi. Virus titers were determined by plaque assay. IL-6 and IFN-β levels in cell supernatants were analyzed by ELISA. Data represent mean ± SD. *P < 0.05 and **P < 0.01 as compared between the indicated groups (Student’s t test). Data are representative of at least two independent experiments. (C) Control (293T-Control), FAF1 overexpressing (293T-FAF1) NLRX1 knockdown (293T-si-NLRX1) and NLRX1 knockdown/FAF1 overexpressing (293T-si-NLRX1/FAF1) HEK293T cells were infected with VSV-GFP (MOI = 0.001), and GFP expression was visualized under a fluorescence microscopy (200 × magnification) and quantified using a fluorescence modulator at 12 hpi. Virus titers were determined by plaque assay. IL-6 and IFN-β levels in cell supernatants were analyzed by ELISA. Data represent mean ± SD. *P < 0.05 and **P < 0.01 as compared between the indicated groups (Student’s t test). Data are representative of at least two independent experiments.
(PDF)
