# Correction: FAS-associated factor-1 positively regulates type I interferon response to RNA virus infection by targeting NLRX1

**DOI:** 10.1371/journal.ppat.1007302

**Published:** 2018-09-21

**Authors:** Jae-Hoon Kim, Min-Eun Park, Chamilani Nikapitiya, Tae-Hwan Kim, Md Bashir Uddin, Hyun-Cheol Lee, Eunhee Kim, Jin Yeul Ma, Jae U. Jung, Chul-Joong Kim, Jong-Soo Lee

The authors would like to correct Fig 5. In Fig 5A, the β-actin panel was duplicated from the β-actin panel in Fig 5B. Additionally, in Fig 5B, the Phospho-TBK1 panel was edited incorrectly. These errors occurred during composition of the final figure and the authors now provide corrected version of Fig 5.

**Fig 5:**
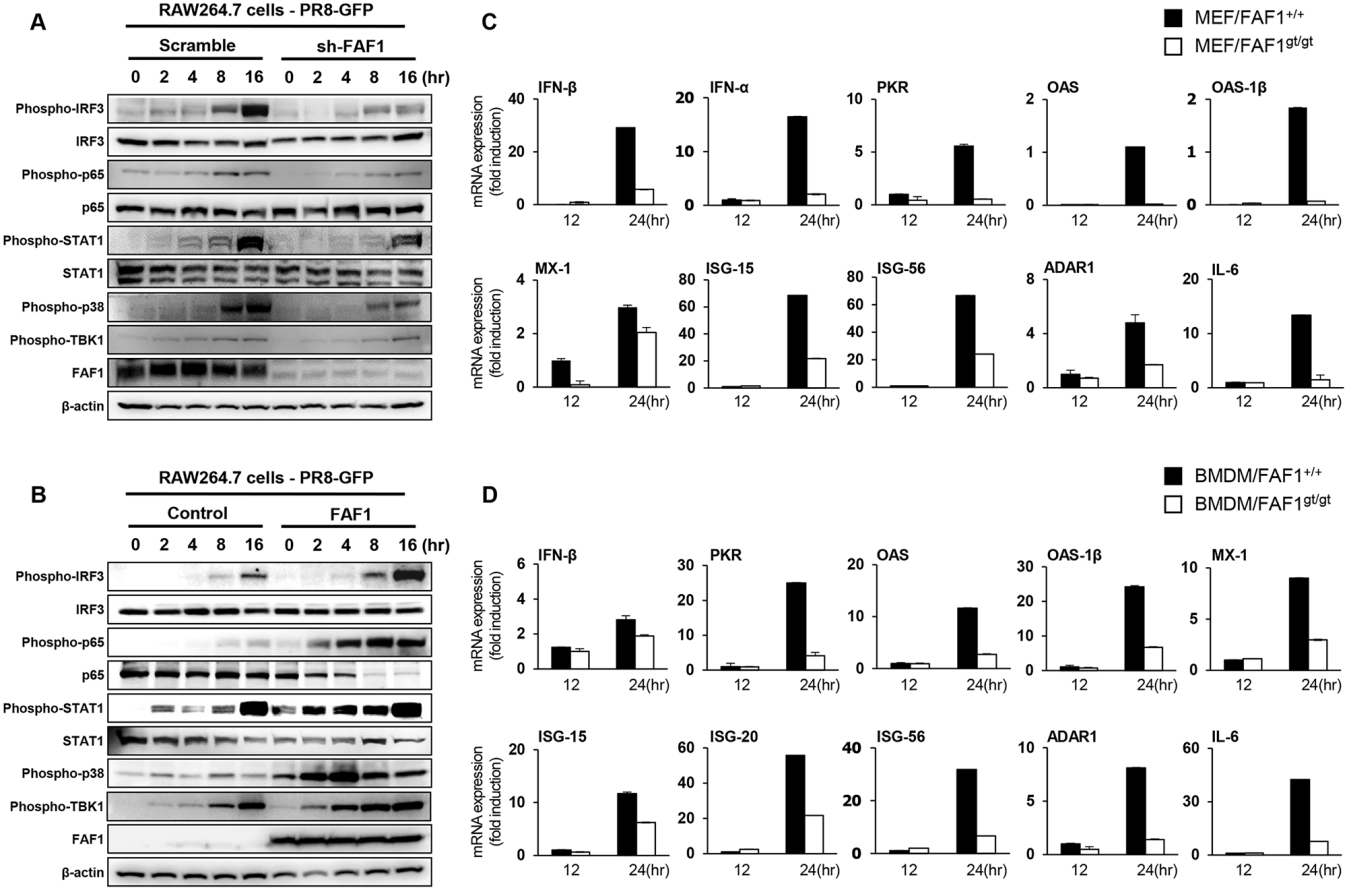
FAF1 activates the type I IFN signaling pathway and induces IFN-related gene expression. (A and B) Control RAW264.7 (RAW-Scramble) and FAF1 knockdown RAW264.7 (RAW-sh-FAF1) cells (A) or control RAW264.7 (RAW-Control) and FAF1-overexpressing RAW264.7 (RAW-FAF1) cells (B) were infected with PR8-GFP (MOI = 2). At the indicated time points after infection, phosphorylated IRF3, p65, STAT1, p38 and TBK1, and total IRF3, p65 and STAT1 were measured in cell extracts by immunoblotting. β-actin was used to confirm equal loading of proteins. (C and D) Wild-type MEFs (MEF/FAF1^+/+^) and FAF1 knockdown MEFs (MEF/FAF1^gt/gt^) (C) and BMDMs isolated from FAF1^+/+^ (BMDM/FAF1^+/+^) and FAF1^gt/gt^ (BMDM/FAF1^gt/gt^) mice (D) were infected with PR8-GFP (MOI = 1 and 3, respectively) for 12 hr, followed by total RNA extraction. Expression of mRNA encoding IFN-β, IFN-α, PKR, OAS, OAS-1β, MX-1, ISG-15, ISG-56, ADAR1 and IL-6 for MEFs and IFN-β, PKR, OAS, OAS-1β, MX-1, ISG-15, ISG-20, ISG-56, ADAR1 and IL-6 for BMDMs was analyzed by qRT-PCR. Data are presented as the mean ± SEM. Data are representative of at least two independent experiments.

The authors confirm that these changes do not alter their findings. The authors have provided raw, uncropped blots as Supporting Information.

## Supporting information

S1 Fig Raw data for Fig 5A. Control RAW264.7 (Scramble) and FAF1 knockdown RAW264.7 (sh-FAF1) cells were infected with PR8-GFP (MOI = 2). At the indicated time points after infection, β-actin were measured in cell extracts by immunoblotting to confirm equal loading of proteins.
(PPTX)

S2 Fig Raw data for Fig 5B. Control RAW264.7 (Control) and FAF1-overexpressing RAW264.7 (FAF1) cells were infected with PR8-GFP (MOI = 2). At the indicated time points after infection, phosphorylated TBK1 was measured in cell extracts by immunoblotting.
(PPTX)

## References

[1] Kim J-H, Park M-E, Nikapitiya C, Kim T-H, Uddin MB, Lee H-C, et al (2017) FAS-associated factor-1 positively regulates type I interferon response to RNA virus infection by targeting NLRX1. PLoS Pathog 13(5): e1006398 10.1371/journal.ppat.1006398 28542569 PMC5456407

