# Supplementary material for: FAS-associated factor-1 positively regulates type I interferon response to RNA virus infection by targeting NLRX1
Source: PLoS Pathog. 2017 May 22;13(5):e1006398. doi: 10.1371/journal.ppat.1006398 (PMC5456407; doi:10.1371/journal.ppat.1006398)
Supplement: S1 Fig — (A and C) The genotypes of the wild-type (FAF1+/+) and FAF1 knockdown (FAF1gt/gt) mice were conducted by generating PCR fragments from tail DNA (A) and from isolated organs (lung, liver, spleen, large intestine and small intestine) (B). PCR fragments were generated with the primers (C) of WT FAF1 allele from FAF1+/+ mice and trapped FAF1 allele from FAF1gt/gt mice. Mouse GAPDH primers were used as an internal reference gene (positive control). (D) FAF1+/+ (n = 4) and FAF1gt/gt (n = 4) mice whole organs (spleen, lung, liver and brain) were collected at 24 hpi of VSV-Indiana (2 × 10^8^ pfu/mouse) via tail-vein injection. The viral load in supernatants of homogenized organs were measured by qRT-PCR. Data represent mean ± SD. *P < 0.05 and **P < 0.01 as compared between the indicated groups (Student’s t test). (E) FAF1+/+ (n = 5) and FAF1gt/gt (n = 5) mice were injected with Poly (I:C) (200 μg per mouse) via tail-vein injection. Sera were collected from the mice at indicated time points and IL-6 and IFN-β were measured by ELISA. Data represent mean ± SD. **P < 0.01 as compared between the indicated groups (Student’s t test). (PDF) [file ppat.1006398.s001.pdf]

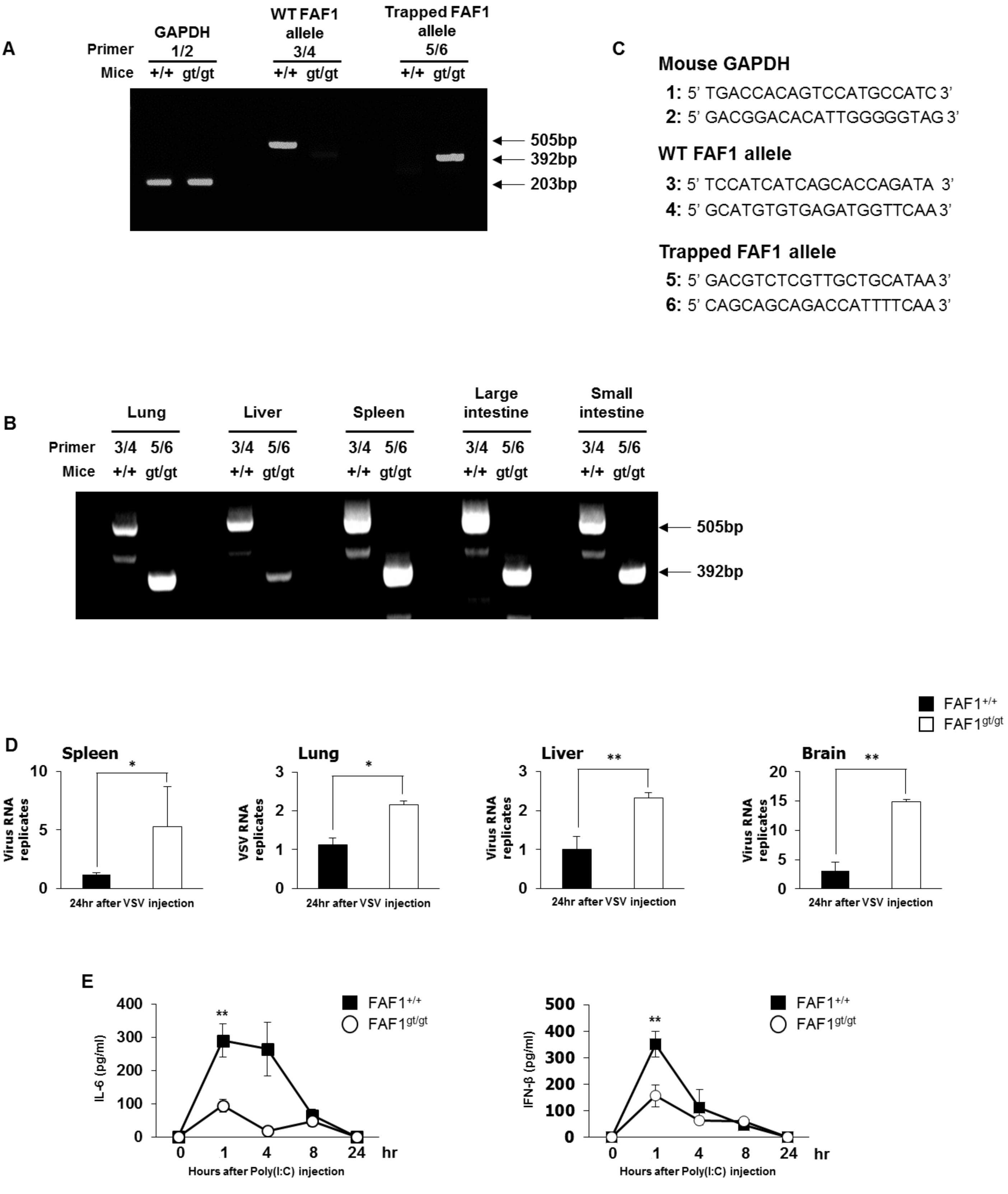

S1 Fig. Immune responses were decreased in FAF1$^{gt/gt}$ mice upon VSV-Indiana infection and Poly (I:C) treatment. (A and C) The genotypes of the wild-type (FAF1$^{+/+}$) and FAF1-knockdown (FAF1$^{gt/gt}$) mice were conducted by generating PCR fragments from tail DNA (A) and from isolated organs (lung, liver, spleen, large intestine and small intestine) (B). PCR fragments were generated with the primers (C) of WT FAF1 allele from FAF1$^{+/+}$ mice and trapped FAF1 allele from FAF1$^{gt/gt}$ mice. Mouse GAPDH primers were used as an internal reference gene (positive control). (D) FAF1$^{+/+}$ (n=4) and FAF1$^{gt/gt}$ (n=4) mice whole organs (spleen, lung, liver and brain) were collected at 24 hpi of VSV-Indiana (2×10^8 pfu/mouse) via tail-vein injection. The viral load in supernatants of homogenized organs were measured by qRT-PCR. Data represent mean ± SD. *$P < 0.05$ and **$P < 0.01$ as compared between the indicated groups (Student's t test). (E) FAF1$^{+/+}$ (n=5) and FAF1$^{gt/gt}$ (n=5) mice were injected with Poly (I:C) (200 μg per mouse) via tail-vein injection. Sera were collected from the mice at indicated time points and IL-6 and IFN-β were measured by ELISA. Data represent mean ± SD. **$P < 0.01$ as compared between the indicated groups (Student's t test).
