# Supplementary material for: FAS-associated factor-1 positively regulates type I interferon response to RNA virus infection by targeting NLRX1
Source: PLoS Pathog. 2017 May 22;13(5):e1006398. doi: 10.1371/journal.ppat.1006398 (PMC5456407; doi:10.1371/journal.ppat.1006398)
Supplement: S2 Fig — (A and B) Wild-type BMDCs (BMDC/FAF1+/+) or FAF1 knockdown BMDCs (BMDC/FAF1gt/gt) were incubated with VSV-GFP (MOI = 2), PR8-GFP (MOI = 3), or Poly (I:C) (20 μg/ml). (C and D) Wild-type (PBMC/FAF1+/+) and FAF1 knockdown PBMCs (PBMC/FAF1gt/gt) were infected with VSV-GFP (MOI = 2). Virus titers were measured by plaque assay (A and C) and qRT-PCR (c). IL-6 and IFN-β levels were evaluated by ELISA (B and D). Data represent mean ± SD. *P < 0.05, **P < 0.01 and ***P < 0.001 as compared between the indicated groups (Student’s t test). (PDF) [file ppat.1006398.s002.pdf]

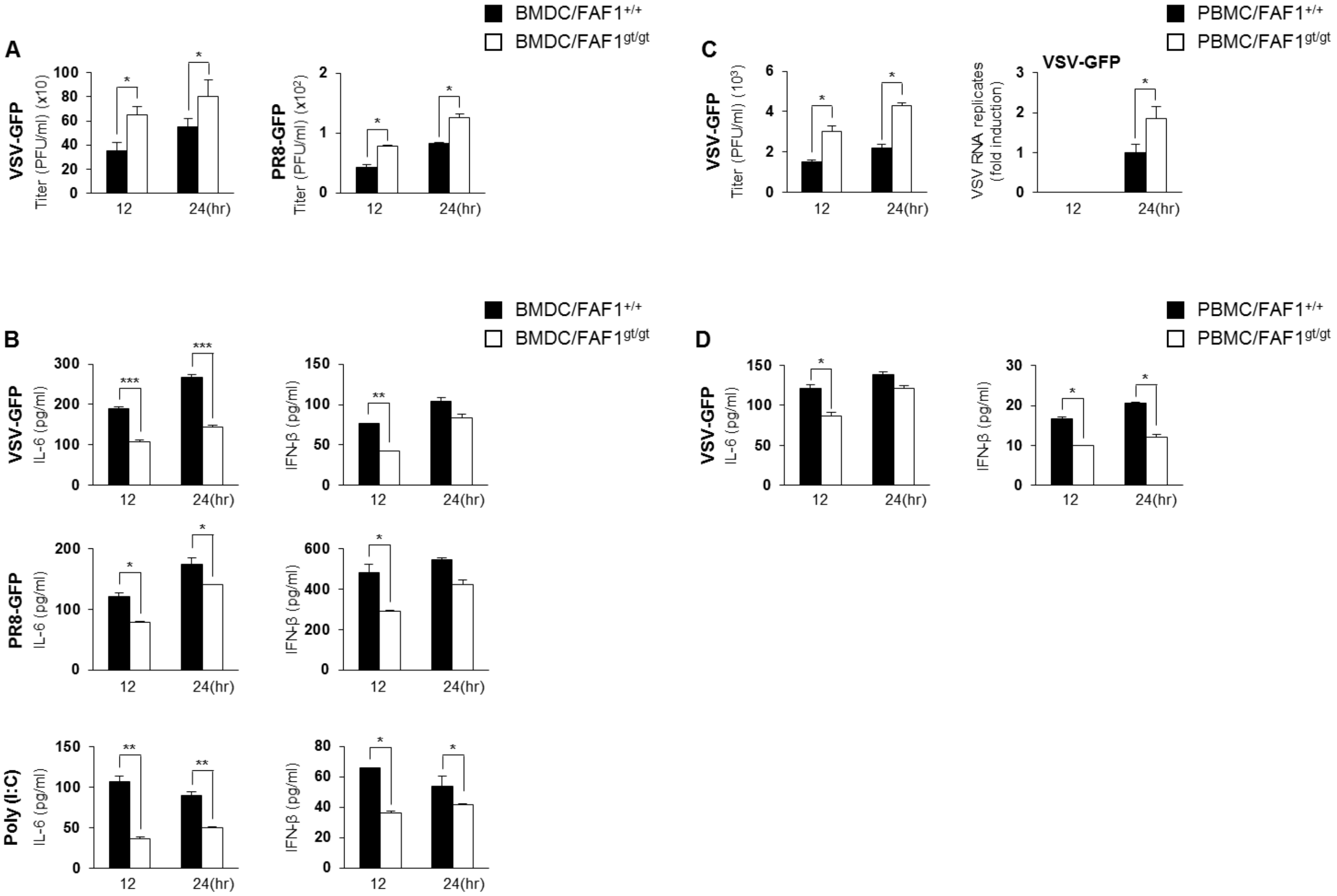

S2 Fig. BMDCs and PBMCs isolated from FAF1$^{gt/gt}$ mice showed high virus replication and low cytokine (IL-6 and IFN-β) secretion against virus infection. (A and B) Wild-type BMDCs (BMDC/FAF1$^{+/+}$) or FAF1 knockdown BMDCs (BMDC/FAF1$^{gt/gt}$) were incubated with VSV-GFP (MOI=2), PR8-GFP (MOI=3), or Poly (I:C) (20 µg/ml). (C and D) Wild-type (PBMC/FAF1$^{+/+}$) and FAF1 knockdown PBMCs (PBMC/FAF1$^{gt/gt}$) were infected with VSV-GFP (MOI=2). Virus titers were measured by plaque assay (A and C) and qRT-PCR (c). IL-6 and IFN-β levels were evaluated by ELISA (B and D). Data represent mean ± SD. **P* < 0.05, ***P* < 0.01 and ****P* < 0.001 as compared between the indicated groups (Student's t test).
