# Supplementary material for: FAS-associated factor-1 positively regulates type I interferon response to RNA virus infection by targeting NLRX1
Source: PLoS Pathog. 2017 May 22;13(5):e1006398. doi: 10.1371/journal.ppat.1006398 (PMC5456407; doi:10.1371/journal.ppat.1006398)
Supplement: S3 Fig — (A) Confirmation of FAF1 protein levels in wild-type MEFs (MEF/FAF1+/+) and FAF1 knockdown MEFs (MEF/FAF1gt/gt) by immunoblot analysis. (B) GFP expression levels of MEF/FAF1+/+ and MEF/FAF1gt/gt infected with NDV-GFP were visualized at 24 hpi, under fluorescence microscopy (200 × magnification), and quantified using a fluorescence modulator. Virus titers were determined by plaque assay. Data represent mean ± SD. **P < 0.01 as compared between the indicated groups (Student’s t test). (C) IL-6 and IFN-β levels in cell supernatants harvested from MEF/FAF1+/+ and MEF/FAF1gt/gt/FAF1 were measured by ELISA at 12 and 24 hpi of NDV-GFP. Data represent mean ± SD. **P < 0.01 and ***P < 0.001 as compared between the indicated groups (Student’s t test). (D) Reconstitution of FAF1 was evaluated by analyzing the levels of FAF1-V5 and β-actin in MEF/FAF1gt/gt and FAF1 reconstituted MEF/FAF1gt/gt (MEF/FAF1gt/gt/FAF1) by immunoblot analysis. β-actin was used to confirm equal protein loading. (E and F) MEF/FAF1gt/gt and MEF/FAF1gt/gt/FAF1 were infected with VSV-GFP (MOI = 0.5), PR8-GFP (MOI = 1) or NDV-GFP (MOI = 1). GFP expression was visualized at 24 hpi, under fluorescence microscopy (200 × magnification), and quantified using a fluorescence modulator. Virus titers were measured by plaque assay (E). Data represent mean ± SD. *P < 0.05, **P < 0.01 and ***P < 0.001 as compared between the indicated groups (Student’s t test). Levels of IL-6 and IFN-β in cell supernatants were assayed by ELISA at 12 and 24 hpi (F). Data represent mean ± SD. *P < 0.05, **P < 0.01 and ***P < 0.001 as compared between the indicated groups (Student’s t test). (G) MEF/FAF1gt/gt and MEF/FAF1gt/gt/FAF1 were treated with Poly (I:C) (20 μg/ml) or 5’ppp-dsRNA (1 μg/ml), and levels of IL-6 and IFN-β in cell supernatants were assayed by ELISA after 12 or 24 hr of treatment. Data represent mean ± SD. *P < 0.05, **P < 0.01 and ***P < 0.001 as compared between the indicated groups (Student’s t test). (PDF) [file ppat.1006398.s003.pdf]

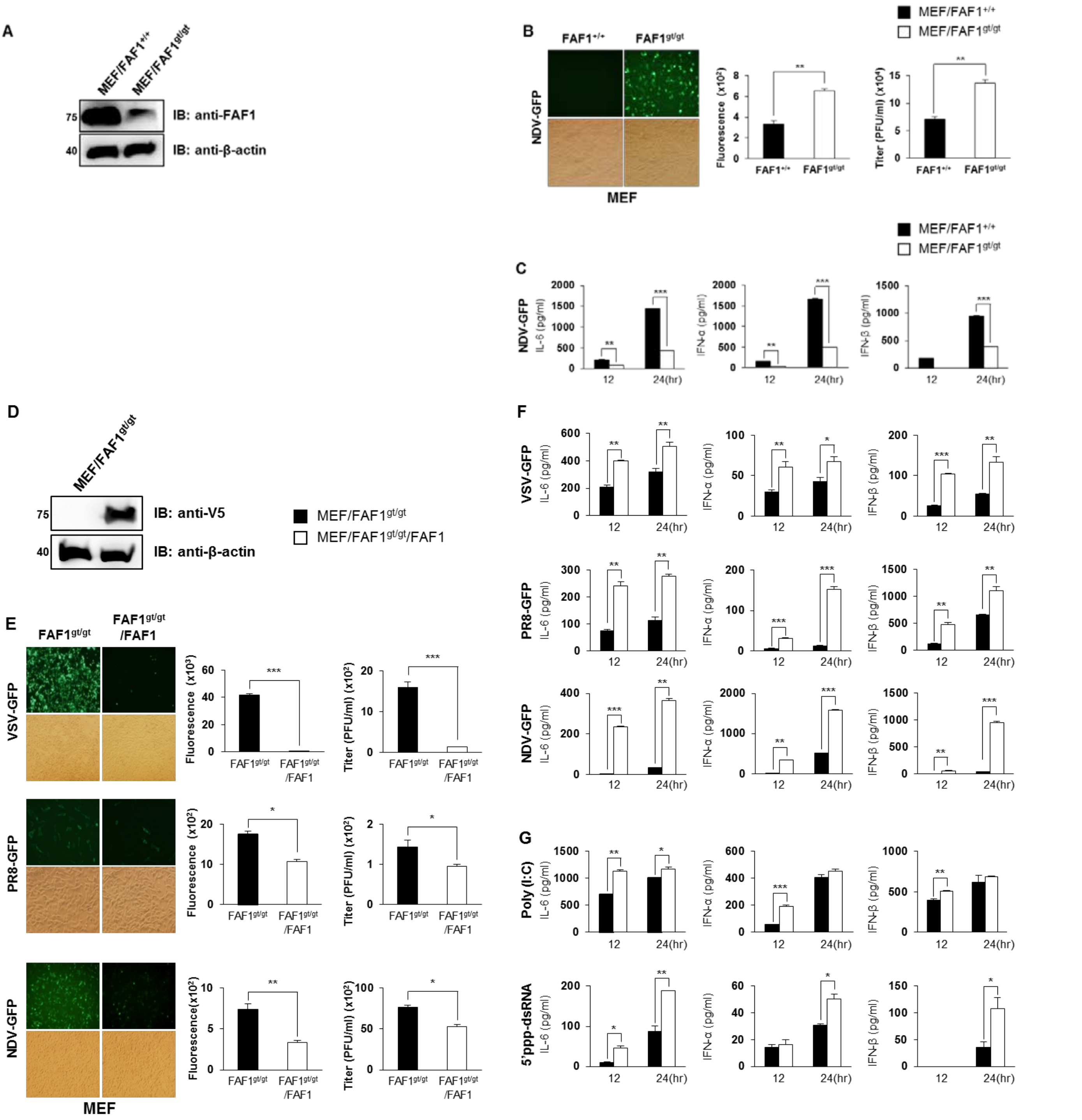

S3 Fig. Knockdown of FAF1 inhibited the immune responses but restored after reconstitution of FAF1 in MEFs. (A) Confirmation of FAF1 protein levels in wild-type MEFs (MEF/FAF1$^{+/+}$) and FAF1 knockdown MEFs (MEF/FAF1$^{gt/gt}$) by immunoblot analysis. (B) GFP expression levels of MEF/FAF1$^{+/+}$ and MEF/FAF1$^{gt/gt}$ infected with NDV-GFP were visualized at 24 hpi, under fluorescence microscopy (200 × magnification), and quantified using a fluorescence modulator. Virus titers were determined by plaque assay. Data represent mean ± SD. ***P* < 0.01 as compared between the indicated groups (Student's t test). (C) IL-6 and IFN-β levels in cell supernatants harvested from MEF/FAF1$^{+/+}$ and MEF/FAF1$^{gt/gt}$/FAF1 were measured by ELISA at 12 and 24 hpi of NDV-GFP. Data represent mean ± SD. ***P* < 0.01 and ****P* < 0.001 as compared between the indicated groups (Student's t test). (D) Reconstitution of FAF1 was evaluated by analyzing the levels of FAF1-V5 and β-actin in MEF/FAF1$^{gt/gt}$ and FAF1 reconstituted MEF/FAF1$^{gt/gt}$ (MEF/FAF1$^{gt/gt}$/FAF1) by immunoblot analysis. β-actin was used to confirm equal protein loading. (E and F) MEF/FAF1$^{gt/gt}$ and MEF/FAF1$^{gt/gt}$/FAF1 were infected with VSV-GFP (MOI=0.5), PR8-GFP (MOI=1) or NDV-GFP (MOI=1). GFP expression was visualized at 24 hpi, under fluorescence microscopy (200 × magnification), and quantified using a fluorescence modulator. Virus titers were measured by plaque assay (E). Data represent mean ± SD. **P* < 0.05, ***P* < 0.01 and ****P* < 0.001 as compared between the indicated groups (Student's t test). Levels of IL-6 and IFN-β in cell supernatants were assayed by ELISA at 12 and 24 hpi (F). Data represent mean ± SD. *P < 0.05, **P < 0.01 and ***P < 0.001 as compared between the indicated groups (Student's t test). (G) MEF/FAF1$^{gt/gt}$ and MEF/FAF1$^{gt/gt}$/FAF1 were treated with Poly (I:C) (20 µg/ml) or 5'ppp-dsRNA (1 µg/ml), and levels of IL-6 and IFN-β in cell supernatants were assayed by ELISA after 12 or 24 hr of treatment. Data represent mean ± SD. **P* < 0.05, ***P* < 0.01 and ****P* < 0.001 as compared between the indicated groups (Student's t test).
