# Supplementary material for: FAS-associated factor-1 positively regulates type I interferon response to RNA virus infection by targeting NLRX1
Source: PLoS Pathog. 2017 May 22;13(5):e1006398. doi: 10.1371/journal.ppat.1006398 (PMC5456407; doi:10.1371/journal.ppat.1006398)
Supplement: S4 Fig — (A) Confirmation of FAF1 protein levels in control RAW264.7 (RAW-Scramble), FAF1 shRNA knockdown RAW264.7 (RAW-shRNA-FAF1) and FAF1 siRNA knockdown RAW264.7 (RAW-siRNA-FAF1) cells by immunoblot analysis. β-actin was used to confirm equal protein loading. (B and C) RAW-Scramble and RAW-siRNA-FAF1 were infected with VSV-GFP (MOI = 1), and GFP expression was visualized under a fluorescence microscopy (200 × magnification) and quantified using a fluorescence modulator at 12 and 24 hpi. Virus titers were determined by plaque assay (B). Data represent mean ± SD. ***P < 0.001 as compared between the indicated groups (Student’s t test). IFN-β levels in cell supernatants were analyzed by ELISA (C). Data represent mean ± SD. *P < 0.05 and **P < 0.01 as compared between the indicated groups (Student’s t test). (D) Confirmation of FAF1 protein levels in control THP-1 (THP-1-Scramble) and FAF1 knockdown THP-1 (THP-1-siRNA-FAF1) by immunoblot analysis. (E and F) THP-1-Scramble and THP-1-siRNA-FAF1 were infected with VSV-GFP (MOI = 1), and GFP expression was visualized under a fluorescence microscopy (200 × magnification) and quantified using a fluorescence modulator at 12 and 24 hpi. Virus titers were determined by plaque assay (E). Data represent mean ± SD. *P < 0.05 and **P < 0.01 as compared between the indicated groups (Student’s t test). IFN-β levels in cell supernatants were analyzed by ELISA (F). Data represent mean ± SD. *P < 0.05 and **P < 0.01 as compared between the indicated groups (Student’s t test). (G) THP-1-Scramble and THP-1-siRNA-FAF1 were treated with Poly (I:C) (20 μg/ml), and levels of IFN-β in cell supernatants were assayed by ELISA. Data represent mean ± SD. **P < 0.01 as compared between the indicated groups (Student’s t test). (PDF) [file ppat.1006398.s004.pdf]

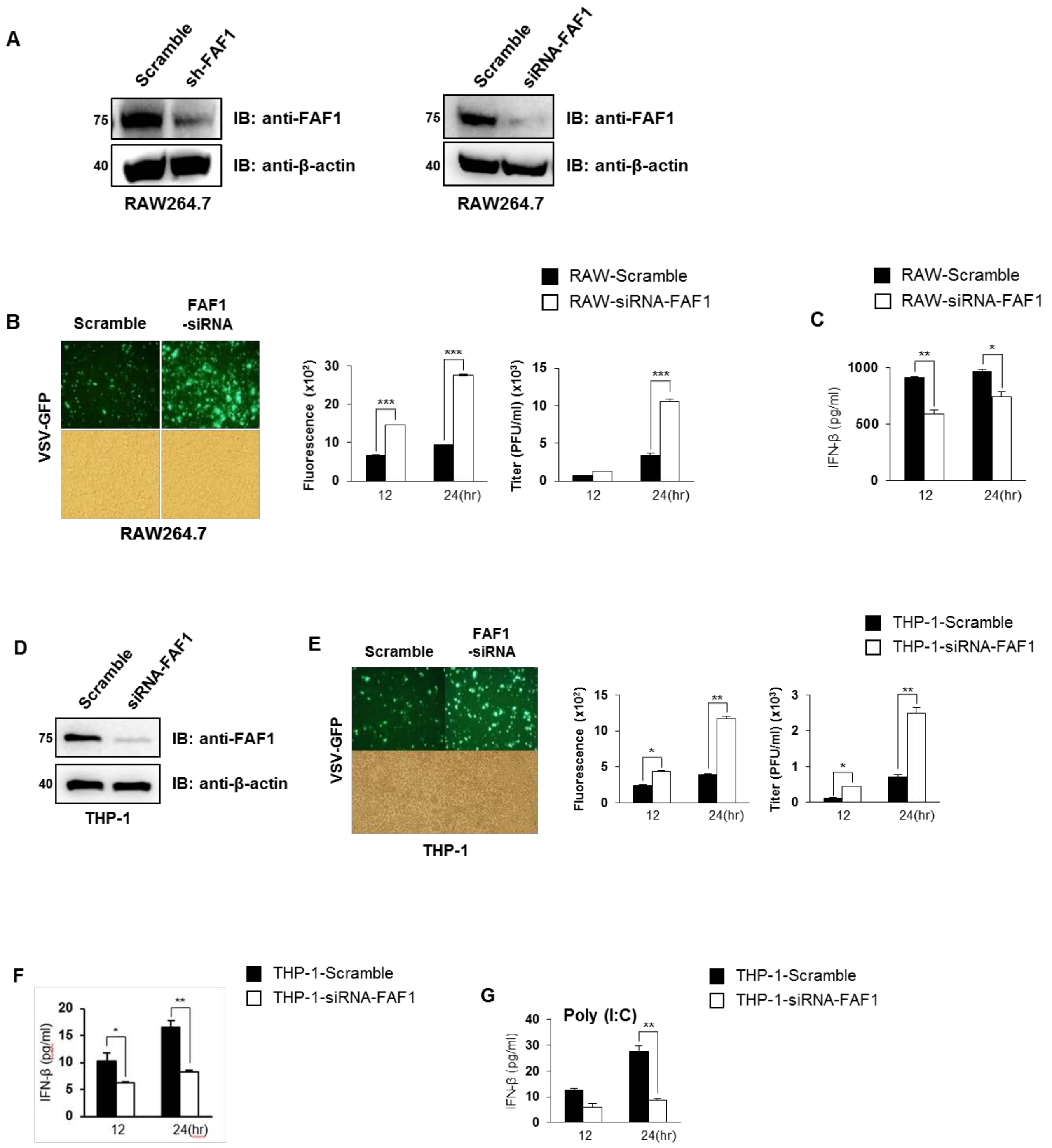

S4 Fig. Knockdown of FAF1 negatively regulated type I IFN secretion against virus infection in RAW264.7 and THP-1 cells. (A) Confirmation of FAF1 protein levels in control RAW264.7 (RAW-Scramble), FAF1 shRNA knockdown RAW264.7 (RAW-shRNA-FAF1) and FAF1 siRNA knockdown RAW264.7 (RAW-siRNA-FAF1) cells by immunoblot analysis. β-actin was used to confirm equal protein loading. (B and C) RAW-Scramble and RAW-siRNA-FAF1 were infected with VSV-GFP (MOI=1), and GFP expression was visualized under a fluorescence microscopy (200 × magnification) and quantified using a fluorescence modulator at 12 and 24 hpi. Virus titers were determined by plaque assay (B). Data represent mean ± SD. ***$P < 0.001$ as compared between the indicated groups (Student's t test). IFN-β levels in cell supernatants were analyzed by ELISA (C). Data represent mean ± SD. *$P < 0.05$ and **$P < 0.01$ as compared between the indicated groups (Student's t test). (D) Confirmation of FAF1 protein levels in control THP-1 (THP-1-Scramble) and FAF1 knockdown THP-1 (THP-1-siRNA-FAF1) by immunoblot analysis. (E and F) THP-1-Scramble and THP-1-siRNA-FAF1 were infected with VSV-GFP (MOI=1), and GFP expression was visualized under a fluorescence microscopy (200 × magnification) and quantified using a fluorescence modulator at 12 and 24 hpi. Virus titers were determined by plaque assay (E). Data represent mean ± SD. *$P < 0.05$ and **$P < 0.01$ as compared between the indicated groups (Student's t test). IFN-β levels in cell supernatants were analyzed by ELISA (F). Data represent mean ± SD. *$P < 0.05$ and **$P < 0.01$ as compared between the indicated groups (Student's t test). (G) THP-1-Scramble and THP-1-siRNA-FAF1 were treated with Poly (I:C) (20 µg/ml), and levels of IFN-β in cell supernatants were assayed by ELISA. Data represent mean ± SD. **$P < 0.01$ as compared between the indicated groups (Student's t test).
