# Supplementary material for: FAS-associated factor-1 positively regulates type I interferon response to RNA virus infection by targeting NLRX1
Source: PLoS Pathog. 2017 May 22;13(5):e1006398. doi: 10.1371/journal.ppat.1006398 (PMC5456407; doi:10.1371/journal.ppat.1006398)
Supplement: S5 Fig — (A) Confirmation of FAF1 protein levels in control RAW264.7 (RAW-Control) and FAF1 overexpressing RAW264.7 (RAW-FAF1) by immunoblot analysis. Immunoblotting of β-actin was used to confirm equal loading. (B and C) RAW-Control and RAW-FAF1 were infected with NDV-GFP (MOI = 1), and GFP expression was visualized under a fluorescence microscopy (200 × magnification) and quantified using a fluorescence modulator at 24 hpi. Virus titers were determined by plaque assay (B). Data represent mean ± SD. *P < 0.05 and **P < 0.01 as compared between the indicated groups (Student’s t test). IL-6, IFN-α, and IFN-β levels in cell supernatants were analyzed by ELISA (C). Data represent mean ± SD. **P < 0.01 and ***P < 0.001 as compared between the indicated groups (Student’s t test). (D) RAW-Control and RAW-FAF1 were treated with Poly (I:C) (20 μg/ml) or 5’ppp-dsRNA (1 μg/ml), and levels of IL-6, IFN-α, and IFN-β in cell supernatants were assayed by ELISA. Data represent mean ± SD. *P < 0.05, **P < 0.01 and ***P < 0.001 as compared between the indicated groups (Student’s t test). (PDF) [file ppat.1006398.s005.pdf]

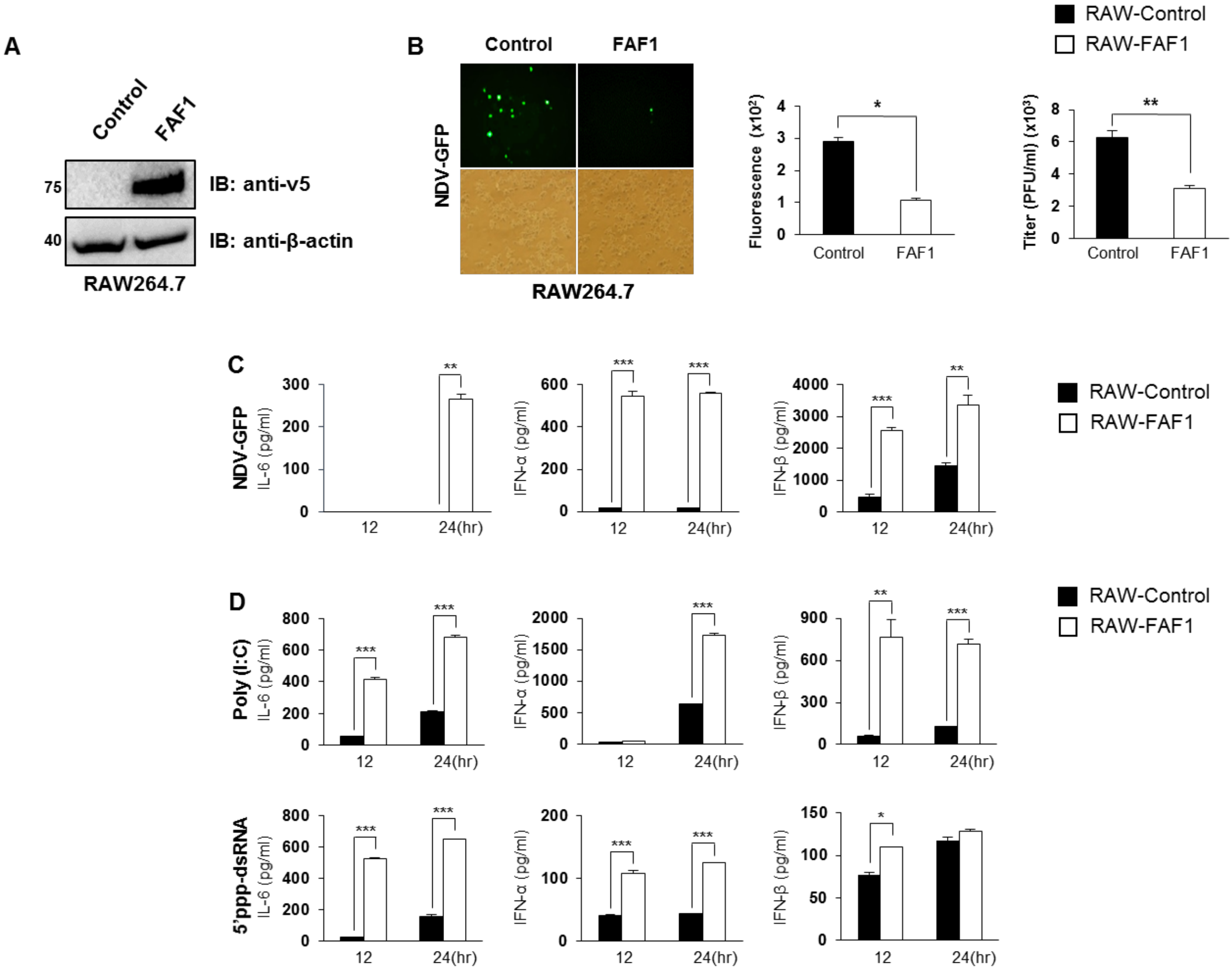

S5 Fig. Overexpression of FAF1 positively regulated type I IFN secretion against virus infection in Raw264.7 cells. (A) Confirmation of FAF1 protein levels in control RAW264.7 (RAW-Control) and FAF1 overexpressing RAW264.7 (RAW-FAF1) by immunoblot analysis. Immunoblotting of β-actin was used to confirm equal loading. (B and C) RAW-Control and RAW-FAF1 were infected with NDV-GFP (MOI=1), and GFP expression was visualized under a fluorescence microscopy (200 × magnification) and quantified using a fluorescence modulator at 24 hpi. Virus titers were determined by plaque assay (B). Data represent mean ± SD. **P* < 0.05 and ***P* < 0.01 as compared between the indicated groups (Student's t test). IL-6, IFN-α, and IFN-β levels in cell supernatants were analyzed by ELISA (C). Data represent mean ± SD. ***P* < 0.01 and ****P* < 0.001 as compared between the indicated groups (Student's t test). (D) RAW-Control and RAW-FAF1 were treated with Poly (I:C) (20 μg/ml) or 5'ppp-dsRNA (1 μg/ml), and levels of IL-6, IFN-α, and IFN-β in cell supernatants were assayed by ELISA. Data represent mean ± SD. **P* < 0.05, ***P* < 0.01 and ****P* < 0.001 as compared between the indicated groups (Student's t test).
