# Supplementary material for: FAS-associated factor-1 positively regulates type I interferon response to RNA virus infection by targeting NLRX1
Source: PLoS Pathog. 2017 May 22;13(5):e1006398. doi: 10.1371/journal.ppat.1006398 (PMC5456407; doi:10.1371/journal.ppat.1006398)
Supplement: S6 Fig — (A) RAW264.7 cells were infected with lentivirus harboring scramble and FAF1 shRNA to prepare control RAW264.7 (RAW-Scramble) and FAF1 knockdown RAW264.7 (RAW-sh-FAF1), respectively. Cells were infected with Adenovirus (MOI = 4). After 24 hr, the virus titer was measured by plaque assay, and IL-6, IFN-α, and IFN-β levels in the supernatant were measured by ELISA at 12 and 24 hpi. Data are presented as the mean ± SEM. Data are representative of at least two independent experiments. (B) Stably expressing control (RAW-Control) and FAF1-overexpressing (RAW-FAF1) cells were infected with VSV-GFP (MOI = 1). At 24 hpi, GFP expression was visualized under a fluorescence microscopy (200 × magnification) and quantified using a fluorescence modulator. Virus titers were measured by plaque assay. Culture supernatants were collected at 12 h and 24 hpi, and IL-6 and IFN-β levels were measured by ELISA. Data are presented as the mean ± SEM. Data are representative of at least two independent experiments. (PDF) [file ppat.1006398.s006.pdf]

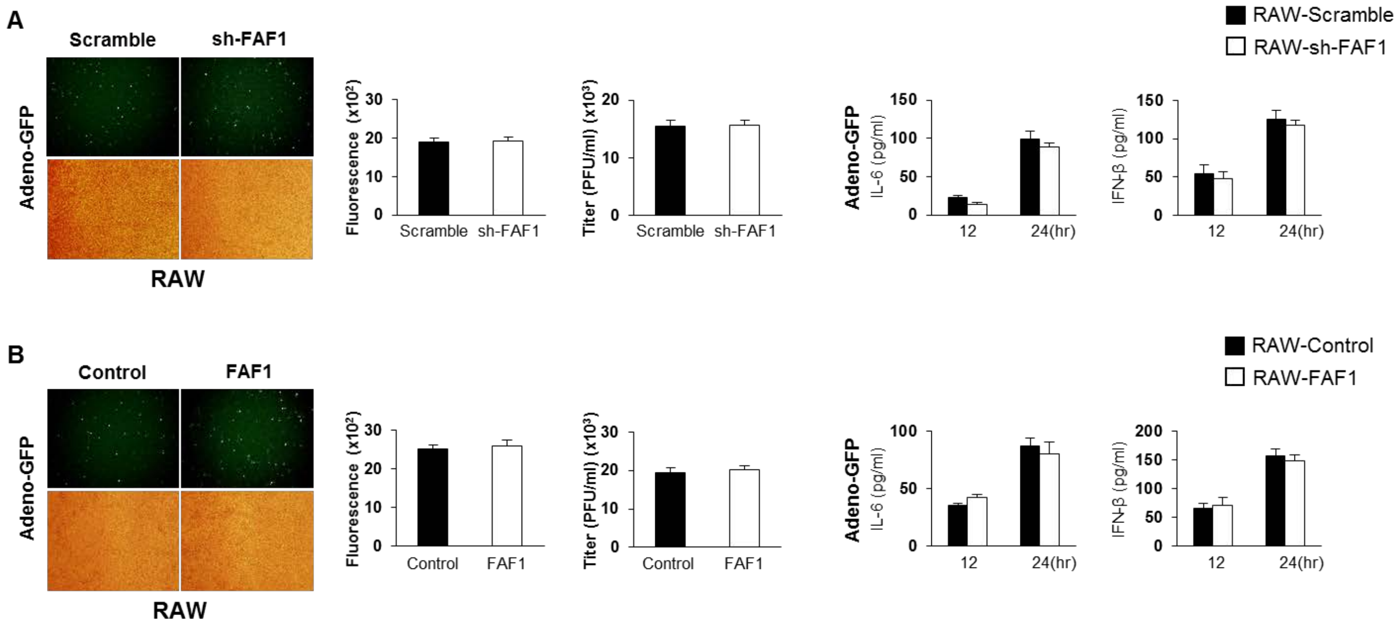

S6 Fig. FAF1 has no role in antiviral activity upon DNA virus infection in RAW264.7 cells.

(A) RAW264.7 cells were infected with lentivirus harboring scramble and FAF1 shRNA to prepare control RAW264.7 (RAW-Scramble) and FAF1 knockdown RAW264.7 (RAW-sh-FAF1), respectively. Cells were infected with Adenovirus (MOI=4). After 24 hr, the virus titer was measured by plaque assay, and IL-6 and IFN-β levels in the supernatant were measured by ELISA at 12 and 24 hpi. Data are presented as the mean ± SEM. Data are representative of at least two independent experiments. (B) Stably expressing control (RAW-Control) and FAF1-overexpressing (RAW-FAF1) cells were infected with VSV-GFP (MOI=1). At 24 hpi, GFP expression was visualized under a fluorescence microscopy (200 × magnification) and quantified using a fluorescence modulator. Virus titers were measured by plaque assay. Culture supernatants were collected at 12 h and 24 hpi, and IL-6 and IFN-β levels were measured by ELISA. Data are presented as the mean ± SEM. Data are representative of at least two independent experiments.
