# Supplementary material for: FAS-associated factor-1 positively regulates type I interferon response to RNA virus infection by targeting NLRX1
Source: PLoS Pathog. 2017 May 22;13(5):e1006398. doi: 10.1371/journal.ppat.1006398 (PMC5456407; doi:10.1371/journal.ppat.1006398)
Supplement: S7 Fig — (A and B) Non-treated or anti-IFNAR antibody treated control RAW264.7 (RAW-Scramble) and FAF1 knockdown RAW264.7 (RAW-sh-FAF1) cells (A) or Wild-type MEFs (MEF/FAF1+/+) and FAF1 knockdown MEFs (MEF/FAF1gt/gt/FAF1) (B) were infected with VSV-GFP (MOI = 1 or 0.5, respectively). After 16 hr, GFP expression was visualized under a fluorescence microscopy (200 × magnification) and quantified using a fluorescence modulator. The virus titer was measured by plaque assay. Data are presented as the mean ± SEM. **P < 0.01 (Student’s t test). Data are representative of at least two independent experiments. (C) Control RAW264.7 (RAW-Scramble) and FAF1 knockdown RAW264.7 (RAW-sh-FAF1) cells were treated with imiquimod (2 μg/ml) or ODN2395 (2 μM). At 12 and 24 hpi, culture supernatants were collected, and IL-6 and IFN-β levels were measured by ELISA. Data are presented as the mean ± SEM. Data are representative of at least two independent experiments. (PDF) [file ppat.1006398.s007.pdf]

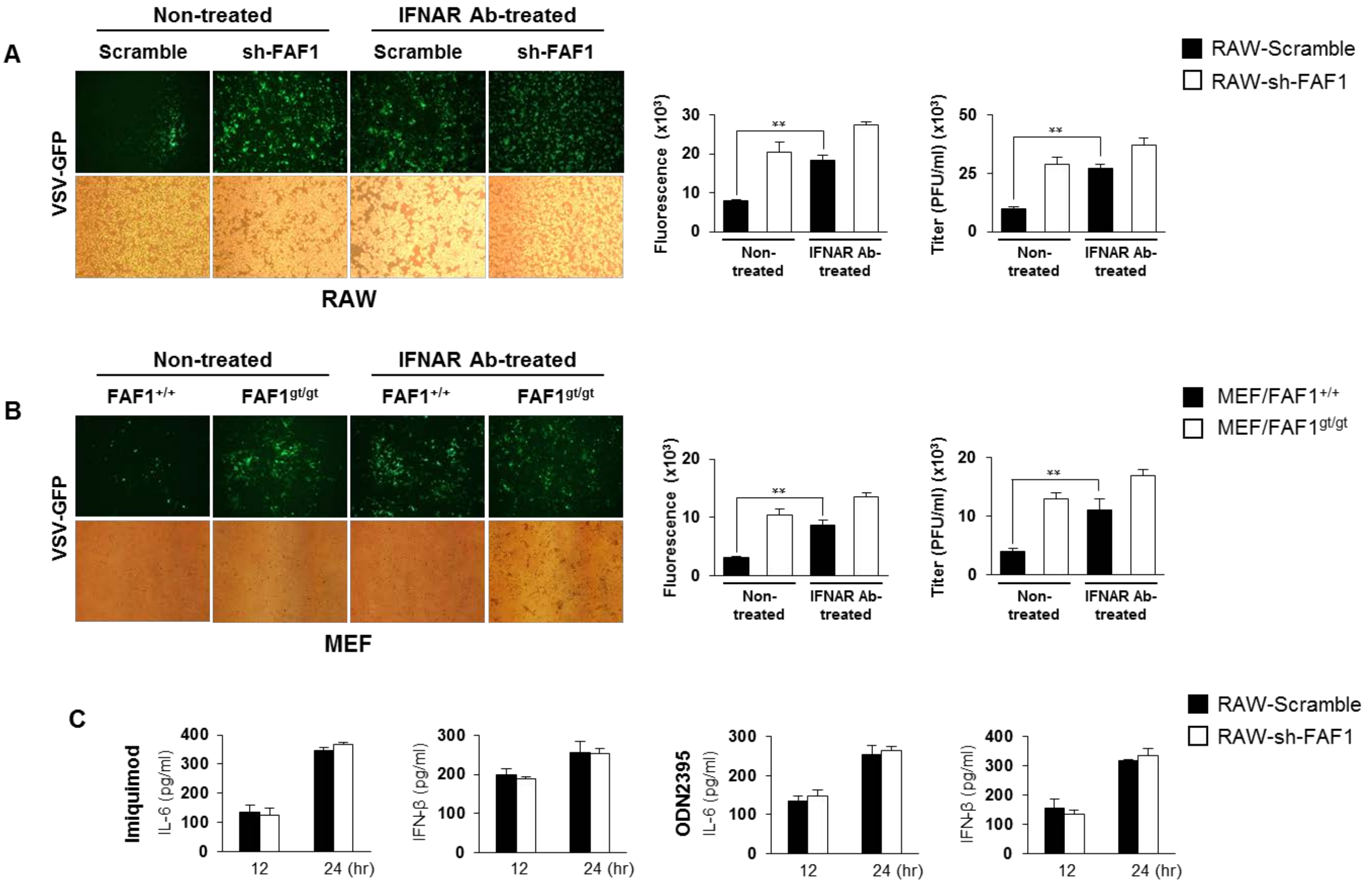

S7 Fig. FAF1 regulate type I IFN signaling through RIG-I-MAVS pathway, and not via TLR7 or TLR9.
(A and B) Non-treated or anti-interferon-α/β receptor (IFNAR) antibody treated control RAW264.7 (RAW-Scramble) and FAF1 knockdown RAW264.7 (RAW-sh-FAF1) cells (A) or Wild-type MEFs (MEF-WT) and FAF1 knockdown MEFs (MEF-FAF1-KD) (B) were infected with VSV-GFP (MOI=1; RAW264.7 cells and MOI=0.5;MEFs). After 16 hr, GFP expression was visualized under a fluorescence microscopy (200 × magnification) and quantified using a fluorescence modulator. The virus titer was measured by plaque assay. Data are presented as the mean ± SEM. **P < 0.01 (Student's t test). Data are representative of at least two independent experiments. (C) Control RAW264.7 (RAW-Scramble) and FAF1 knockdown RAW264.7 (RAW-sh-FAF1) cells were treated with imiquimod (2 μg/ml) or ODN2395 (2 μM). At 12 and 24 hpi, culture supernatants were collected, and IL-6 and IFN-β levels were measured by ELISA. Data are presented as the mean ± SEM. Data are representative of at least two independent experiments.
