# Supplementary material for: FAS-associated factor-1 positively regulates type I interferon response to RNA virus infection by targeting NLRX1
Source: PLoS Pathog. 2017 May 22;13(5):e1006398. doi: 10.1371/journal.ppat.1006398 (PMC5456407; doi:10.1371/journal.ppat.1006398)
Supplement: S8 Fig — (A) The amount of phosphorylated IRF3, p65, STAT1, p38, and TBK1 and total IRF3, p65, and STAT1 was examined by immunoblot analysis of cell extracts from FAF1 knockdown MEFs (MEF/FAF1gt/gt) and FAF1-reconstituted MEF/FAF1gt/gt (MEF/FAF1gt/gt/FAF1) at the indicated times after PR8-GFP (MOI = 3) infection. β-actin was used to confirm equal protein loading. (B and C) MEF/FAF1gt/gt and MEF/FAF1gt/gt/FAF1 (B) and PBMCs isolated from FAF1+/+ (PBMC/FAF1+/+) and FAF1gt/gt (PBMC/FAF1gt/gt) mice (C) were infected with PR8-GFP (MOI = 1 and 3, respectively) for 12 hr. Total RNA was extracted from infected cells to determine the expression of mRNA encoding IFN-β, IFN-α, PKR, OAS, MX-1, ISG-15, ISG-20, ISG-56, ADAR1 and IL-6 for MEFs and IFN-β, PKR, OAS, OAS-1β, ISG-15, ISG-20 and ISG-56 for PBMCs was analyzed by qRT-PCR. Data represent mean ± SD. (PDF) [file ppat.1006398.s008.pdf]

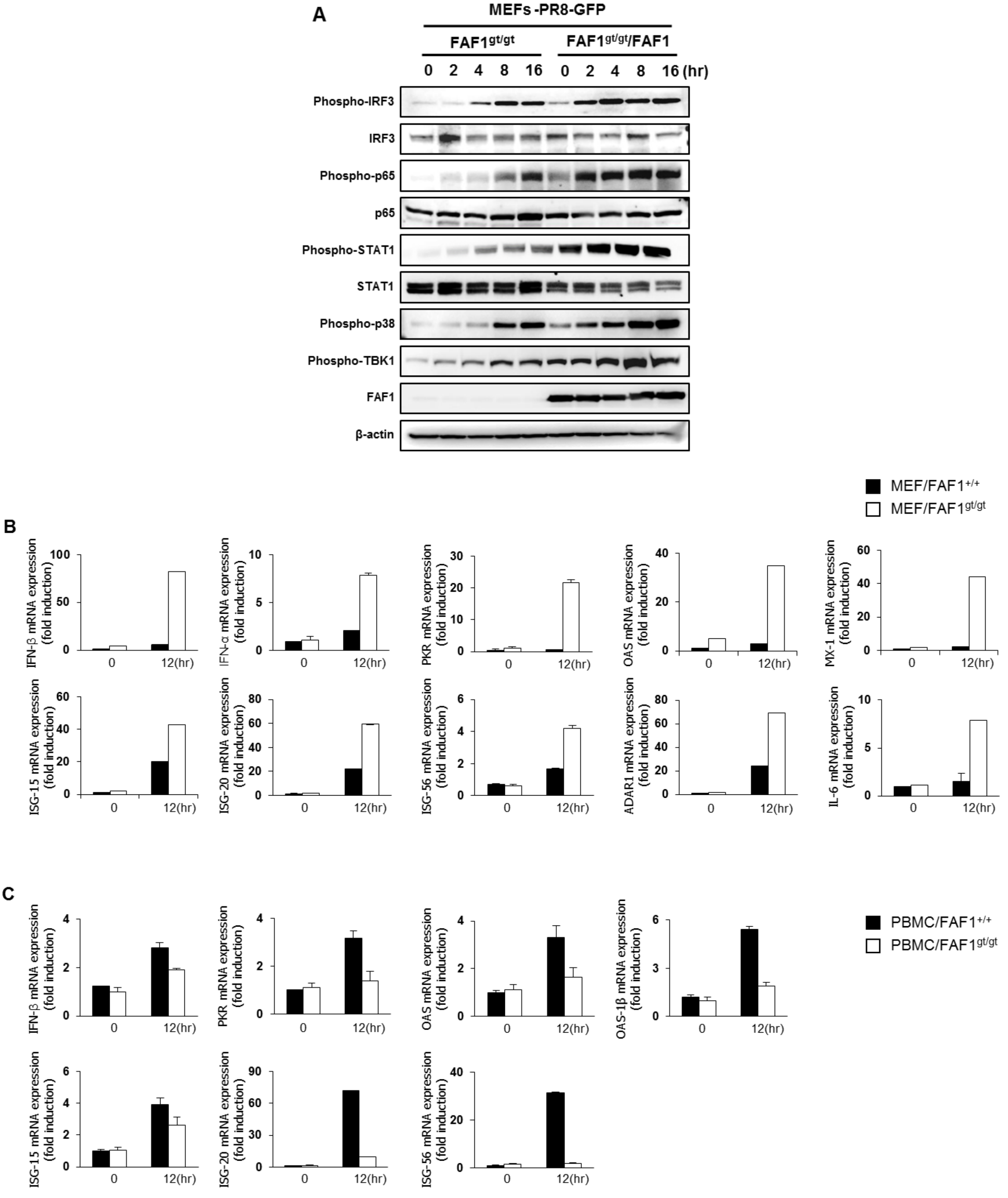

S8 Fig. FAF1 enhances type I IFN signaling and expression of mRNA encoding IFN and IFN-related gene. (A) The amount of phosphorylated IRF3, p65, STAT1, p38, and TBK1 and total IRF3, p65, and STAT1 was examined by immunoblot analysis of cell extracts from FAF1 knockdown MEFs (MEF/FAF1$^{gt/gt}$) and FAF1-reconstituted MEF/FAF1$^{gt/gt}$ (MEF/FAF1$^{gt/gt}$/FAF1) at the indicated times after PR8-GFP (MOI=3) infection. β-actin was used to confirm equal protein loading. (B and C) MEF/FAF1$^{gt/gt}$ and MEF/FAF1$^{gt/gt}$/FAF1 (B) and PBMCs isolated from FAF1$^{+/+}$ (PBMC/FAF1$^{+/+}$) and FAF1$^{gt/gt}$ (PBMC/FAF1$^{gt/gt}$) mice (C) were infected with PR8-GFP (MOI=1 and 3, respectively) for 12 hr. Total RNA was extracted from infected cells to determine the expression of mRNA encoding IFN-β, IFN-α, PKR, OAS, MX-1, ISG-15, ISG-20, ISG-56, ADAR1 and IL-6 for MEFs and IFN-β, PKR, OAS, OAS-1β, ISG-15, ISG-20 and ISG-56 for PBMCs was analyzed by qRT-PCR.. Data represent mean ± SD.
