# Supplementary material for: FAS-associated factor-1 positively regulates type I interferon response to RNA virus infection by targeting NLRX1
Source: PLoS Pathog. 2017 May 22;13(5):e1006398. doi: 10.1371/journal.ppat.1006398 (PMC5456407; doi:10.1371/journal.ppat.1006398)
Supplement: S9 Fig — (A) Different cell types were infected with PR8-GFP (BMDMs, MOI = 3, RAW264.7, MOI = 2; MEFs, MOI = 1; THP-1, MOI = 3; HEK293T, MOI = 1; HeLa, MOI = 2; and A549, MOI = 2). Total RNA was extracted from infected cells at indicated time points and FAF1 mRNA expression was analyzed by qRT-PCR. Data represent mean ± SD. (PDF) [file ppat.1006398.s009.pdf]

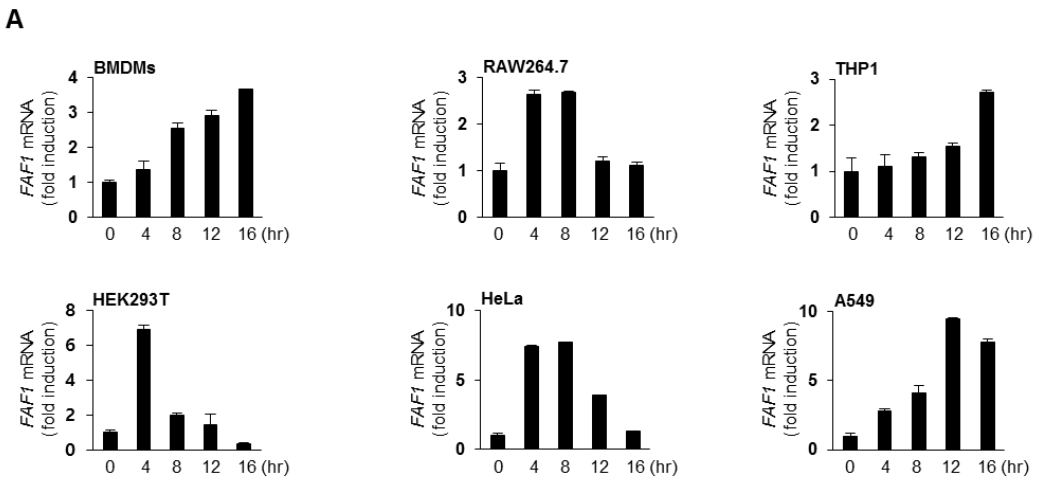

S9 Fig. FAF1 mRNA expression was increased in various cell types after PR8-GFP infection. (A) Different cell types were infected with PR8-GFP (BMDMs, MOI=3, RAW264.7, MOI=2; MEFs, MOI=1; THP-1, MOI=3; HEK293T, MOI=1; HeLa, MOI=2; and A549, MOI=2). Total RNA was extracted from infected cells at indicated time points and FAF1 mRNA expression was analyzed by qRT-PCR. Data represent mean ± SD.
