# Supplementary material for: FAS-associated factor-1 positively regulates type I interferon response to RNA virus infection by targeting NLRX1
Source: PLoS Pathog. 2017 May 22;13(5):e1006398. doi: 10.1371/journal.ppat.1006398 (PMC5456407; doi:10.1371/journal.ppat.1006398)
Supplement: S10 Fig — (A) HEK293T cells were transfected with the indicated GST-NLRX1 constructs (aa 556–975, 75–975 and 1–975) and FAF1-V5. GST pull-down (GST PD) was conducted followed by immunoblot analysis with anti-V5 and anti-GST antibodies. WCL were immunoblotted with anti-V5 and anti-GST antibodies. (PDF) [file ppat.1006398.s010.pdf]

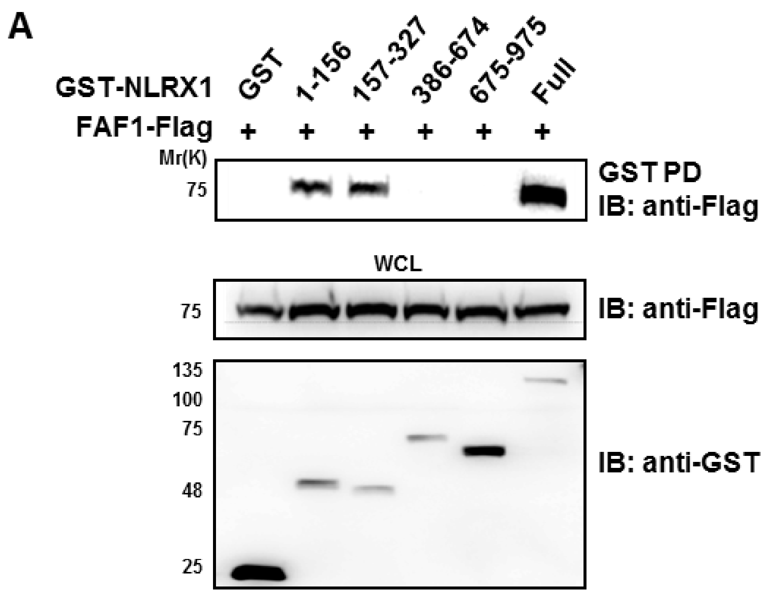

S10 Fig. The amino acid 1-327 region of NLRX1 is responsible for the interaction with FAF1.

(A) HEK293T cells were transfected with the indicated GST-NLRX1 constructs (aa 1-156, 157-.327, 386-674, 675-975) and FAF1-Flag. GST pull-down (GST PD) was conducted followed by immunoblot analysis with an anti-Flag antibody. WCL were immunoblotted with anti-Flag and anti-GST antibodies.
