# Supplementary material for: FAS-associated factor-1 positively regulates type I interferon response to RNA virus infection by targeting NLRX1
Source: PLoS Pathog. 2017 May 22;13(5):e1006398. doi: 10.1371/journal.ppat.1006398 (PMC5456407; doi:10.1371/journal.ppat.1006398)
Supplement: S11 Fig — (A) Confirmation of FAF1 and NLRX1 protein levels in control (Scramble) and FAF1 (siRNA-FAF1) or NLRX1 (siRNA-NLRX1) siRNA knockdown HEK293T cells by immunoblot analysis. β-actin was used to confirm equal protein loading. (B) Control (293T-Control), FAF1 knockdown (293T-si-FAF1) NLRX1 knockdown (293T-si-NLRX1) and NLRX1/FAF1 knockdown (293T-si-NLRX1/si-FAF1) HEK293T cells were infected with VSV-GFP (MOI = 0.001), and GFP expression was visualized under a fluorescence microscopy (200 × magnification) and quantified using a fluorescence modulator at 12 hpi. Virus titers were determined by plaque assay. IL-6 and IFN-β levels in cell supernatants were analyzed by ELISA. Data represent mean ± SD. *P < 0.05 and **P < 0.01 as compared between the indicated groups (Student’s t test). Data are representative of at least two independent experiments. (C) Control (293T-Control), FAF1 overexpressing (293T-FAF1) NLRX1 knockdown (293T-si-NLRX1) and NLRX1 knockdown/FAF1 overexpressing (293T-si-NLRX1/FAF1) HEK293T cells were infected with VSV-GFP (MOI = 0.001), and GFP expression was visualized under a fluorescence microscopy (200 × magnification) and quantified using a fluorescence modulator at 12 hpi. Virus titers were determined by plaque assay. IL-6 and IFN-β levels in cell supernatants were analyzed by ELISA. Data represent mean ± SD. *P < 0.05 and **P < 0.01 as compared between the indicated groups (Student’s t test). Data are representative of at least two independent experiments. (PDF) [file ppat.1006398.s011.pdf]

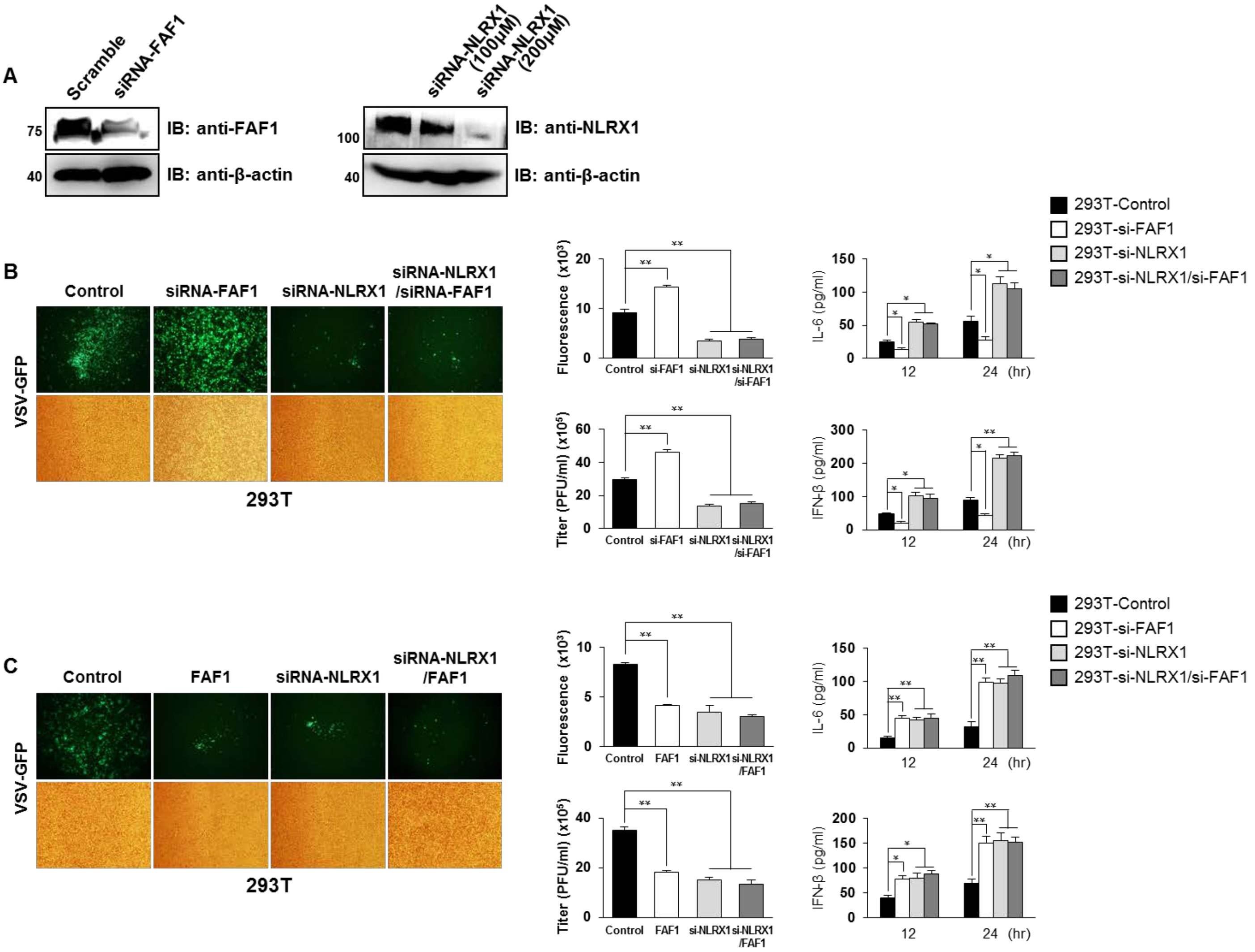

S11 Fig. Knockdown of NLRX1 abrogate FAF1 mediated antiviral effect of type I IFN in HEK293T cells. (A) Confirmation of FAF1 and NLRX1 protein levels in control (Scramble) and FAF1 (siRNA-FAF1) or NLRX1 (siRNA-NLRX1) siRNA knockdown HEK293T cells by immunoblot analysis. β-actin was used to confirm equal protein loading. (B) Control (293T-Control), FAF1 knockdown (293T-si-FAF1) NLRX1 knockdown (293T-si-NLRX1) and NLRX1/FAF1 knockdown (293T-si-NLRX1/si-FAF1) HEK293T cells were infected with VSV-GFP (MOI=0.001), and GFP expression was visualized under a fluorescence microscopy (200 × magnification) and quantified using a fluorescence modulator at 12 hpi. Virus titers were determined by plaque assay. IL-6 and IFN-β levels in cell supernatants were analyzed by ELISA. Data represent mean ± SD. **P* < 0.05 and ***P* < 0.01 as compared between the indicated groups (Student's t test). Data are representative of at least two independent experiments. (C) Control (293T-Control), FAF1 overexpressing (293T-FAF1) NLRX1 knockdown (293T-si-NLRX1) and NLRX1 knockdown/FAF1 overexpressing (293T-si-NLRX1/FAF1) HEK293T cells were infected with VSV-GFP (MOI=0.001), and GFP expression was visualized under a fluorescence microscopy (200 × magnification) and quantified using a fluorescence modulator at 12 hpi. Virus titers were determined by plaque assay. IL-6 and IFN-β levels in cell supernatants were analyzed by ELISA. Data represent mean ± SD. *P < 0.05 and **P < 0.01 as compared between the indicated groups (Student's t test). Data are representative of at least two independent experiments.
