# Supplementary figures and images for: Correction: FAS-associated factor-1 positively regulates type I interferon response to RNA virus infection by targeting NLRX1
Source: PLoS Pathog. 2018 Sep 21;14(9):e1007302. doi: 10.1371/journal.ppat.1007302 (PMC6150512; doi:10.1371/journal.ppat.1007302)

## Slide 1
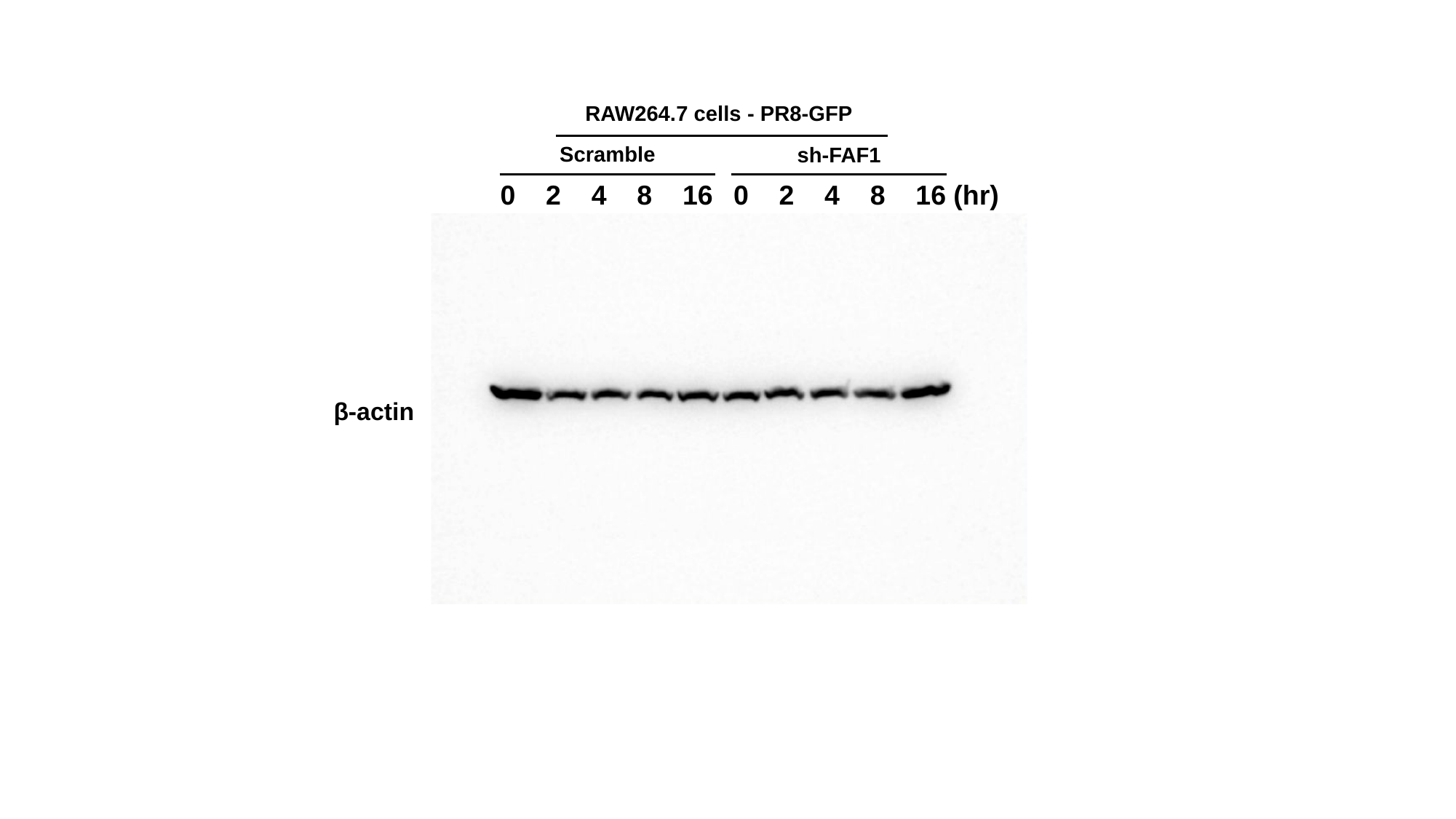

RAW264.7 cells - PR8-GFP
Scramble
sh-FAF1
0 2 4 8 16
0 2 4 8 16 (hr)
β-actin

Supplement: S1 Fig — Control RAW264.7 (Scramble) and FAF1 knockdown RAW264.7 (sh-FAF1) cells were infected with PR8-GFP (MOI = 2). At the indicated time points after infection, β-actin were measured in cell extracts by immunoblotting to confirm equal loading of proteins. (PPTX) [file ppat.1007302.s001.pptx]

## Slide 1
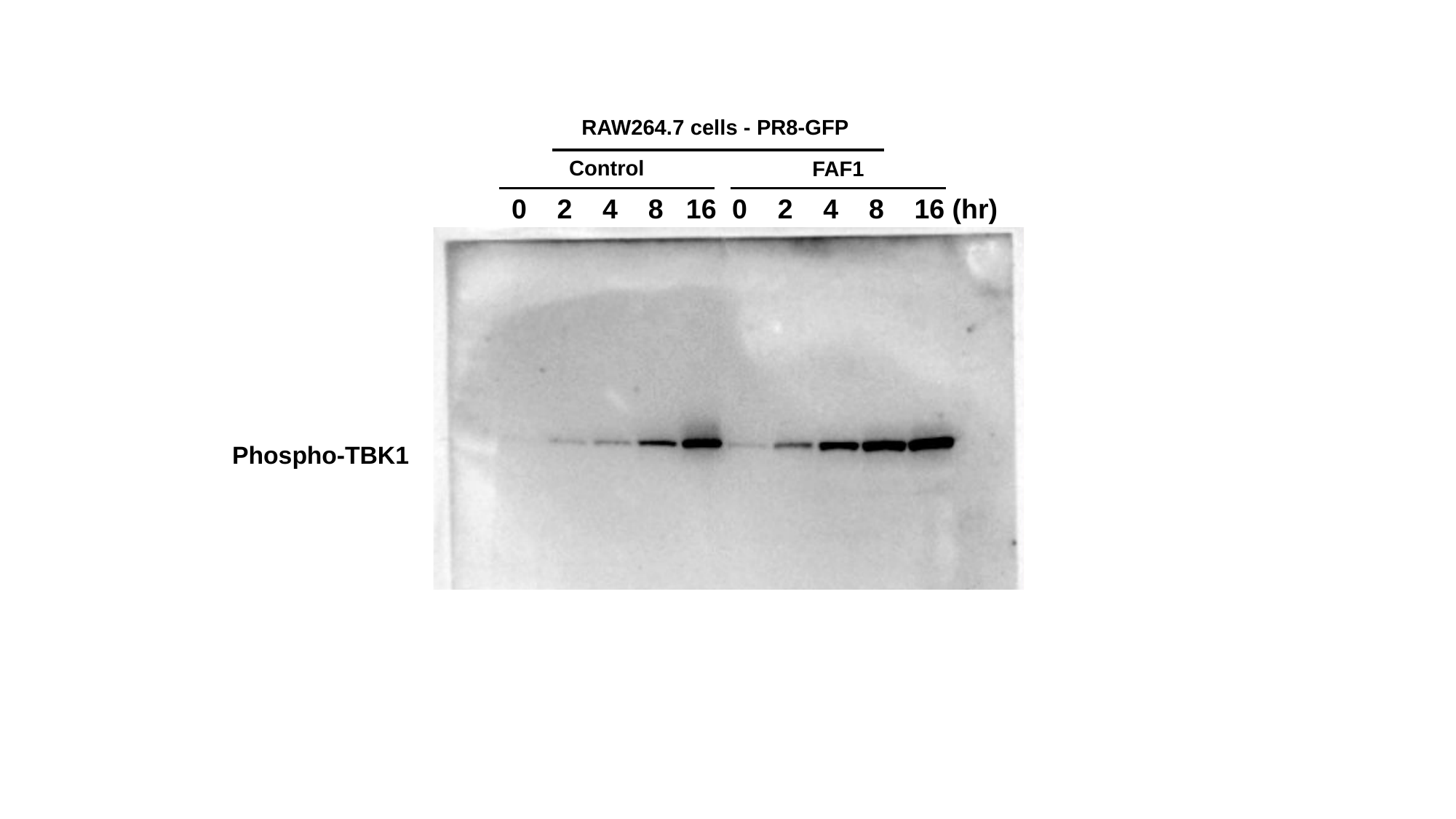

RAW264.7 cells - PR8-GFP
Control
FAF1
0 2 4 8 16
0 2 4 8 16 (hr)
Phospho-TBK1

Supplement: S2 Fig — Control RAW264.7 (Control) and FAF1-overexpressing RAW264.7 (FAF1) cells were infected with PR8-GFP (MOI = 2). At the indicated time points after infection, phosphorylated TBK1 was measured in cell extracts by immunoblotting. (PPTX) [file ppat.1007302.s002.pptx]
